# Transcriptome characterisation and population genetics of *Cunninghamiakonishii* Hayata – An endangered gymnosperm and implication for its conservation in Vietnam

**DOI:** 10.3897/BDJ.13.e153663

**Published:** 2025-07-18

**Authors:** Bich Hong Ha, Mai Phuong Pham, Quoc Khanh Nguyen, Thi Tuyet Xuan Bui, Giap Dinh Vu, Syed Noor Muhammad Shah, Dinh Duy Vu

**Affiliations:** 1 College of Forestry Biotechnology, Vietnam National University of Forestry, Hanoi, Vietnam College of Forestry Biotechnology, Vietnam National University of Forestry Hanoi Vietnam; 2 Joint Vietnam–Russia Tropical Science and Technology Research Center, Hanoi, Vietnam Joint Vietnam–Russia Tropical Science and Technology Research Center Hanoi Vietnam; 3 Institute of Biology, Vietnam Academy of Science and Technology, Hanoi, Vietnam Institute of Biology, Vietnam Academy of Science and Technology Hanoi Vietnam; 4 HaUI Institute of Technology, Hanoi University of Industry (HaUI), Hanoi, Vietnam HaUI Institute of Technology, Hanoi University of Industry (HaUI) Hanoi Vietnam; 5 Department of Horticulture, Gomal University, Dera Ismail Khan, Pakistan Department of Horticulture, Gomal University Dera Ismail Khan Pakistan

**Keywords:** *Cunninghamiakonishii* Hayata, genetic diversity, gymnosperms, Southeast Asia, EST-SSR

## Abstract

Biodiversity loss and degradation activities have a significant impact and devastating consequences on the ecosystem, eventually posing a major threat to many plant species, including *Cunninghamiakonishii*. Deforestation and the growth of settlements are the main factors that affect the biodiversity hotspots in Vietnam such as Northwest, Northeast, North Central and Central Highlands regions. This has led to a decline of the species, so effective conservation strategies are urgently required. This study aimed to identify simple sequence repeat markers within expressed sequence tags from *C.konishii*, develop markers from them and assess the potential of those markers for diversity and population structure analysis of the plant. The Illumina HiSeq™ 4000 sequencing technology was applied for the transcriptomic analysis of *C.konishii* and genetic differentiation and population structure of *C.konishii* in Vietnam. In this study, the transcriptomes of *C.konishii* were analysed using the Illumina HiSeq^TM^ 4000 sequencing system and a total of 5,361,856,500 base pairs were generated. *De novo* assembly indicated that 58,905 unigenes were generated (average length = 736.4 bp, N50 = 1,869 bp, Q20 = 98.44% and Q30 = 95.08%). A total of 23,232 and 16,510 unigenes had significant similarities amongst Nr and Swiss-Prot, respectively. In the GO database, 12,056 (20.47%) unigenes were annotated and these genes were divided into three major categories and 50 subcategories. In the KOG analysis, 13,248 (22.49 %) unigenes were annotated and divided into 25 gene function categories. In the KEGG analysis, 8,714 (14.79%) unigenes were annotated. According to the related pathways involved, they could be classified into 56 subclasses. In this study, we have identified a total of 2,854 EST-SSRs markers. Of the 960 primer pairs, 99 were validated and reported for polymorphism. The genetic diversity within and amongst *C.konishii* populations was studied using 10 SSR markers. A sample size of 96 trees considered from three distant populations in Vietnam was analysed in this study. Our data determined *PIC* = 0.67, *Na* = 4.05, *Ne* = 2.76 and *P* = 100%. We reported moderate levels of genetic diversity with *Ho* = 0.56 and *He* = 0.58 and the fixation index value was recorded as positive for three populations (XL, HSP and PH). The bottleneck tests showed clear evidence of a bottleneck in PH population sizes. Genetic differentiation amongst populations was recorded very low (*F_ST_* = 0.029), indicating gene flow (*Nm* = 8.169). This result indicates gene exchange between the populations of ancient *C.konishii* from different geographical areas and regions. The analysis of molecular variance (AMOVA) showed that high genetic variation existed within individuals (90.68%) compared to amongst populations (2.97%). A Discriminant Analysis of Principal Components (DAPC) and Bayesian clustering grouped the populations into three genetically similar clusters. Additionally, candidate genes related to essential oil biosynthesis were identified. This study provides the first EST-SSR marker-based genetic diversity and population structure analysis of *C.konishii*, offering valuable insights for breeding and conservation efforts. The findings establish a key genetic resource for future conservation strategies with the aim of preserving this endangered species.

## Introduction

*Cunninghamiakonishii* Hayata is an essential timber species distributed mainly in four provinces of Vietnam, namely Ha Giang, Son La, Thanh Hoa and Nghe An (Pham and Vu 2022) and extends to Taiwan (China) and Laos (Farjon 2010). Morphologically, *C.konishii* is a 40–50 m tall tree with monopodial growth. The trunk diameter reaches 3-4.5 m at breast height (DBH) on full maturity. It can be found at elevations 1300 - 2800 m in Taiwan (Liang 2010), 900-2200 m in Laos (Averyanov et al. 2014) and 1000-1600 m in Vietnam (Phan et al. 2017). Both the number and size of *C.konishii* naturally occurring populations in Vietnam are severely restricted (Phan et al. 2017) as its timber is one of the best building materials known for its fragrance (essential oil) and outstanding durability (Tran et al. 2015). Its current commercial exploitation level is very high, but cultivation is rare (Farjon 2010). There is a gradual decline in the natural population of *C.konishii* due to the destruction of the primary forests. This species is assessed as "Endangered" by the IUCN and listed on the IUCN Red List (Thomas and Yang 2013) and as endangered (EN) in the Vietnam Red Data Book 2024 (Most and Vast 2024). The complete genetics of the species remain unexplored due to the limitation of molecular marker availability. Therefore, it is of prime importance to study its distribution, ecology, natural active components, phytochemicals and other potential resources to ensure the survival of the species.

A simple sequence repeat (SSR) marker is a convenient tool in genetic diversity, population structure and evolution of plants because of its co-dominance, polymorphism and higher stability (Varshney et al. 2005). These microsatellites or SSRs consist of tandem repeat motifs of few base pairs in length, but may vary in number at a given locus and are widely distributed throughout the eukaryotic genomes (Srivastava et al. 2019). Currently, microsatellites are popular due to their multi-allelic nature, co-dominant inheritance, polymorphic, ease of detection by polymerase chain reaction and extensive genome coverage (Ueno et al. 2012). Microsatellites are also species-specific markers (Zane et al. 2002, Squirrell et al. 2003) and ideal for population structure and genetic diversity studies since they exhibit co-dominance and high level of reproducibility (Ellis and Burke 2007, Haji et al. 2014). The conventional strategy of developing SSRs through the screening of partial genomic libraries is a time-consuming, laborious and expensive process. With recent progress in bioinformatics, an alternative approach can be used other than conventional because databases can be mined for SSRs development (Kantety et al. 2002, Kumpatla and Mukhopadhyay 2005). In recent years, with the increase in quantities of sequences of genomes in the National Centre for Biotechnology Information (NCBI) database, there has also been recorded an increase in the number of expressed sequence tags (ESTs). These EST-derived SSR markers are an alternative complement to existing genomic SSRs, which can be developed in silico by data mining of EST databases. EST–SSRs have several intrinsic advantages over genomic SSRs.They are more transferable across taxonomic levels, distributed, found in coding sequences and may be related to the function of potential genes (Yu and Li 2008). EST–SSRs have been widely used in genotypes diversity (Eujayl et al. 2002, Chabane et al. 2005), genetic mapping (Luro et al. 2008), population genetic analysis (Kim and Sappington 2013) and phylogenetic studies (Stàgel et al. 2008).

The next-generation sequencing (NGS) technology, particularly high throughput in transcriptome sequencing, has offered extensive expressed sequence data for non-model plants (Egan et al. 2012, Vlk and Řepková 2017). Transcriptome sequencing is a simple and effective tool used to develop a large number of unigene-based microsatellites (SSRs) for comprehensive analysis of the genome in plants (Zhang et al. 2021). Transcriptome sequence generally represents the genetic information of gene expression and may be directly related to the gene function (Jain 2011). Recently, the development of SSR markers, based on transcriptome sequences, has been reported in many conifer species such as *Taxusflorinii*, *T.wallichiana*, *T.mairei*, *Glyptostrobuspensilis*, *Torreyagrandis*, *Amentotaxusargotaenia*, *Pinuskoraiensis*, *Abiesalba Fokieniahodginsii Platycladusorientalis* (Liu et al. 2011, Zhu and Lou 2012, Postolache et al. 2013, Wu et al. 2017, Qin et al. 2017, Ding et al. 2017, Du et al. 2017, Zeng et al. 2018, Li et al. 2019, Ruan et al. 2019, Li et al. 2020, Götz et al. 2024). In Chinese fir (*C.lancelata*), around 100 pairs of SSR markers developed through transcriptome sequences have been reported (Huang et al. 2012, Wen et al. 2017, Duan et al. 2017, Lin et al. 2020, Götz et al. 2024), but SSR markers development through transcriptome sequences have not been reported in *C.konishii*. The genetics of environmental adaptation in *C.konishii* have not been studied. Therefore, transcriptome sequencing tool needs to be used to understand the genetic and metabolic adaptations of the species to its habitats. The study of genetic diversity within and between the population will help in the efforts to manage and conserve the species. It will contribute to the knowledge of ecology, evolution and genetics for the conservation of the species.

The Illumina HiSeq^TM^4000 platform was used to analyse the transcriptome and develop EST-SSR markers. This will help in understanding of genetic diversity and population structure of the species, which is very important for the conservation of *C.konishii*.

## Materials and Methods

### Sample collection and RNA extraction

**Figure 1.**
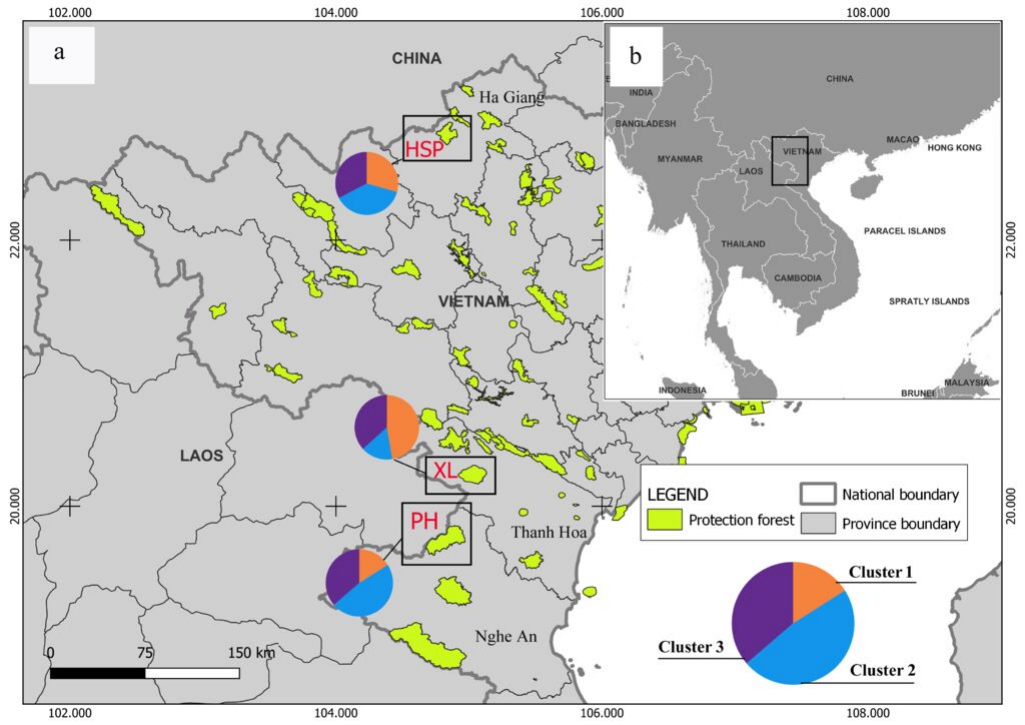
Map of field survey locations and geographic distributions of *C.konishii* in Vietnam. Different symbols show genetic clustering into three clusters, as revealed in population structure analyses, based on microsatellite data. XL, Xuan Lien Nature Reserve; HSP, Hoang Su Phi Nature Reserve; PH, Pu Hoat Nature Reserve.

The leaves, stem and roots of five different native *C.konishii* were collected from Hoang Su Phi Nature Reserve, Ha Giang Province (northern Vietnam, 22°39’49.6” N, 104°45’35.1” E) in February 2025 (Fig. 1). The collected samples were immediately placed in liquid nitrogen and transported to the Laboratory of Molecular Biology, Vietnam-Russia Tropical Centre and stored at -80^o^C for RNA extraction. In order to perform Illumina sequencing, total RNA was first extracted from each sample using a plant RNA kit and then processed with DNase I. These procedures followed the manufacturer's instructions (Omega Biotack, Inc.). The quality of the processed RNA samples was checked by running electrophoresis on 1% agarose gels and an Agilent 2100 Bioanalyzer (Agilent Technologies, CA, USA) and the quantity was checked using a Nanodrop ND-2000 spectrophotometer (NanoDrop Technologies, DE, USA). Finally, equal amounts of RNA from different samples were pooled for RNA sequencing.

### Illumina sequencing and de novo transcriptome assembly

RNA samples were sent to Breeding Biotechnologies Co., Ltd. for mRNA-seq library construction and sequencing using Illumina HiSeq^TM^ 4000 next-generation platform technology (Illumina, Inc., CA, USA). The quality of the raw reads was checked. The adapter contaminants with the raw reads > 5% of unknown nucleotides (N) and > 20% low-quality bases (quality value < 10) were removed to obtain high-quality clean reads. The clean reads were de novo assembled into contigs using TRINITY (Grabherr et al. 2011). The contigs were clustered into unigenes using TGICL v. 2.1 (Pertea et al. 2003).

### Functional annotations

To determine the predicted function, all unigene transcripts were used and compared to the NCBI non-redundant (Nr) protein database (Deng et al. 2006) and Swiss-Prot database (Apweiler 2004), Gene Ontology (GO) (Ashburner et al. 2000), eukaryotic Orthologous Groups of proteins (KOG) (Koonin et al. 2004) and Kyoto Encyclopedia of Genes and Genomes (KEGG) (Kanehisa 2004) databases using BLAST (E-value of 10^-5^) to search for homologues. Sequences were aligned with the Protein family database (Pfam) to obtain unigene annotation information using the HMMER (Finn et al. 2013). BLAST2GO software (Conesa et al. 2005) was used to determine gene ontology (GO) annotations of assembled unigenes for the categories of biological processes and molecular functions of cellular components. The BLAST software aligned the unigene sequences to the eukaryotic Orthologous Groups (KOG) clusters. KEGG pathways annotations were analysed for the metabolic pathways and the related gene functions.

### Primer design

EST–SSRs with appropriate flanking regions were selected for primer design using Primer 3 software (Untergasser et al. 2012). A total of 960 primer pairs were designed and synthesised from Integrated DNA Technologies, Inc. To amplify DNA fragments containing SSRs, the primer designing conditions were 55–65°C melting temperature, 40–60% GC content and a length of PCR product between 100 and 300 base pairs.

### DNA extraction, microsatellite polymorphism identification

Amongst the 100 SSR primer pairs randomly selected for PCR identification, 10 were successfully amplified by 96 *C.konishii* individuals from three natural populations (Fig. 1, Suppl. material 6). The total genomic DNA was extracted from the plant DNA Kit according to the manufacturer's instructions (BioTeke, Beijing, China). The DNA purity and integrity were tested by a Nanodrop ND-2000 spectrophotometer (NanoDrop Technologies, DE, USA) and then diluted to 20 ng‧µl^-1^. The PCR amplification reaction was performed in a 25 µl reaction volume, comprising 2.5 µl of template DNA, 12.5 µl of 2X Taq Master Mix, 1 µl of each primer and 8 µl of deionised water. The GeneAmp PCR System 9700 (Applied Biosystems, USA) was used for the PCR amplification with the following conditions: initial denaturation at 94°C for 3 minutes, 40 cycles at 94°C for 30 s, a primer-specific annealing temperature for 30 s and 70°C for 1 min, followed by a final extension at 72°C for 10 min and then holding the samples at 4°C until they were analysed. The PCR products were sized and relatively quantified between samples on a 5300 Fragment Analyzer (Agilent) with an Agilent DNF-905 dsDNA Kit (1-500 bp) (Agilent).

### Data analysis

**Genetic diversity**

Null alleles and other genotyping errors were detected using the MICRO-CHECKER v.2.0 software (Van Oosterhout et al. 2004), with 1000 bootstrap iterations over loci to generate the expected homozygote and heterozygote frequencies. CERCUS (Kalinowski et al. 2007) was used to estimate each locus' PIC (polymorphism information content) value. Variables for genetic diversity per loci and population, including the number of alleles (Na), number of effective alleles (*Ne*), number of private alleles (*Np*), the observed heterozygosities (*Ho*), the expected heterozygosities (*He*), inbreeding coefficient (*Fis*) and gene flow (*Nm*) were calculated using the programme GenAlEx 6.5 (Peakall and Smouse 2012). The individual inbreeding model was performed to evaluate the F_IS_ index for null allele frequency (F_IS_IIM) using INEst (Chybicki and Burczyk 2008). Deviations from Hardy-Weinberg equilibrium (HWE) for loci within populations were tested in Cercus (Kalinowski et al. 2007), based on 1,000 permutations of alleles amongst individuals. With the use of BOTTLENECK v.1.2, each population was checked for deviations in heterozygosity from the expected values under mutation-drift equilibrium (Piry et al. 1999). Three different mutational and drift models: IAM (the infinite allele model), SMM (the stepwise mutation model) and TPM (the two-phased model of mutation) were considered. For TPM, 70% single-step mutation and 10,000 repeats were used. A one-tailed Wilcoxon signed-rank test was utilised to determine the significance of the findings.

**Genetic differentiation and population structure.**

The F_ST_ (Weir and Cockerham 1984) and G'_ST_ values (Hedrick 2007) were able to quantify the genetic divergence that exists between the populations (GenAlEx 6.5). Using ARLEQUIN v.3.0, the significance of the F_ST_ values in population pairs was assessed at a significance level of 0.05. The results of this testing were considered significant (Excoffier et al. 2005). The analysis of molecular variance (AMOVA) was carried out with the assistance of ARLEQUIN v.3.0, which was used to carry out the significance testing for the variance components involved. The outcomes of 10,000 different permutations served as the basis for this testing. The Mantel matrix correspondence test was used to assess the correlation between genetic distance and geographic distance amongst different geographic populations to test for a relationship between genetic distance and geographic distance (kilometres) amongst populations (Legendre and Fortin 2010). A neighbour-joining (NJ) tree was implemented, based on the Fst values using Poptree2 (Takezaki et al. 2010). To undertake an analysis of the population structure using STRUCTURE v.2.3.4, the Bayesian clustering method was put into action (Pritchard et al. 2000). Establishing the admixture model with correlated allele frequencies required ten distinct runs for each number of groups in the dataset (K). These runs were carried out with 500,000 Markov Chain Monte Carlo (MCMC) iterations and a burn-in time of 100,000 iterations. K ranged from 1 to 15. STRUCTURE HARVESTER (Earl and vonHoldt 2011) was used to detect the number of groups that best fitted the dataset, based on the K as determined by Evanno et al. (2005) to assess the ideal value of K. This was done to determine the optimal value of K. Then the duplicated findings were aligned with the help of CLUMPP v.1.1.2 (Jakobsson and Rosenberg 2007) and the allocated cluster membership bar plots were created with DISTRUCT v.1.1. (Rosenberg 2003). A Discriminant Analysis of Principal Components (DAPC) was also performed using the Adegenet package in R v.4.0.2 programme to discover clusters of individuals with a common genetic ancestor (Jombart et al. 2010). The DAPC was implemented without any previous information on the population's origin. The Bayesian information criterion (BIC) was used to establish the "ideal" cluster size and the number of clusters, denoted by the letter "K", ranged from one to twenty in the experiments. The DAPC was also responsible for performing the previous information analysis to determine how individuals should be assigned to populations. To obtain a better visual representation of the genetic clusters, the complot function of Adegenet was included. The *xval*DAPC function was used to keep the top fourteen principal components of the principal component analysis (98.5% of the variance that was conserved), as well as the seven discriminant eigenvalues.

## Results

### Illumina sequencing and de novo assembly

Through RNA-seq, 17,872,855 original sequences of *C.konishii* were obtained with 5,361,856,500 base pairs, Q20 (98.44%) and Q30 (95.08%). The number of ‘N” bases (ambiguous base) and GC-content were 0 and 44.45%, respectively. It shows that the high-throughput sequencing platform has obtained a higher quantity and quality of *C.konishii* sequences, which is conducive to the subsequent data assembly and meets the needs of later bioinformatics research. After the clean reads were assembled de novo, a total of 99,325 transcripts with sequence information of 105,702,940 bp and 58,905 unigenes with sequence information of 44,965,632 bp were obtained (Table 1). Analysis of the sequence length of the transcript shows that its average length was 1,064 bp and N50 was 1,869 bp. Amongst them, short sequences of 200 to 300 bp were in the majority, with 25,236 sequences accounting for 25.31% of the total; sequences with a length of 300 to 500 bp were 18,932 (19.06%) sequences; sequences of 500 to 1,000 bp in length were 18,346 (18.47%) sequences; 20,870 (21.01%) sequences were ranged from 1,000 to 2,000 bp and sequences longer than or equal to 2,000 bp accounted for 16,041 (16.15%) sequences (Suppl. material 1, Table 1). Unigene analysis and statistical results show that its average length was 763.4 bp, N50 was 1,447 bp, of which the sequences of 200 to 300 bp accounted for 21,752 (36.93%) of the total sequence; 14,338 (24.34%) sequences were between 300 and 500 bp; 9,378 (15.92%) sequences were in 500 to 1,000 bp; 7,987 (13.56%) sequences were in 1001 to 2000 bp; and the sequences of more than 2,000 bp account for 5.450 (9.25%) (Suppl. material 2, Table 1). By processing a large number of sequences obtained by high-throughput RNA-seq, the integrity of unigenes data after assembly is significantly improved and the next step of analysis and statistics were performed.

### Functional annotation and classification of unigenes

A total of 58,905 Unigenes were obtained through BLAST software in 8 major databases (COG, GO, KEGG, KOG, Pfam, Swissprot, eggNOG and Nr) (Table 2). Among them, 23,232 Unigenes were successfully annotated in Nr, accounting for 39.44% of the total number of Unigenes; in KEGG, there were 8,714 successful annotations, accounting for 14.79% of the total; in SwissProt, there were 16,510 successful annotations, accounting for 28.03% of the total; 16,688 successful annotations in Pfam, accounting for 28.33% of the total; 12,056 successful annotations in GO, accounting for the total 20.47% of the total; 13,248 successfully annotated in KOG, accounting for 22.49% of the total; 7,892 successfully annotated in COG, accounting for 13.40% of the total and in eggNOG there are 21,467 successful annotations, accounting for 36.44% of the total. The number of successfully annotated sequences in all 8 major databases was 23,878, accounting for 40.54%.

Through the Nr library's comparison, 23,232 unigenes of *C.konishii* found similar sequences in the Nr database (Suppl. material 3). The annotation matching species mainly include *Piceasitchensis* (6,658; 28.66%), *Amborellatrichopoda* (2,109; 9.08%), *Nelumbonucifera* (1,034; 4.45%), *Macleayacordata* (571; 2.46%), *Physcomitrellapatens* (552; 2.38%), *Marchantiapolymorpha* (491; 2.11%), *Vitisvinifera* (375; 1.61%), *Pinustaeda* (326; 1.4%), *Elaeisguineensis* (319; 1.37%) and *Phoenixdactylifera* (301; 1.30%). A large fraction indicated similarities to genes in other species (10,445; 44.96%). From the annotated information, it can be concluded that most of the sequences of *C.konishii* can be matched in angiosperms. In general, from the distribution of sequence similarity, it can be seen that *C.konishii* has a high matching degree in the Nr database. However, due to the lack of genome and transcriptome information of *C.konishii*, no match in the database was found for some unigenes.

**Figure 2.**
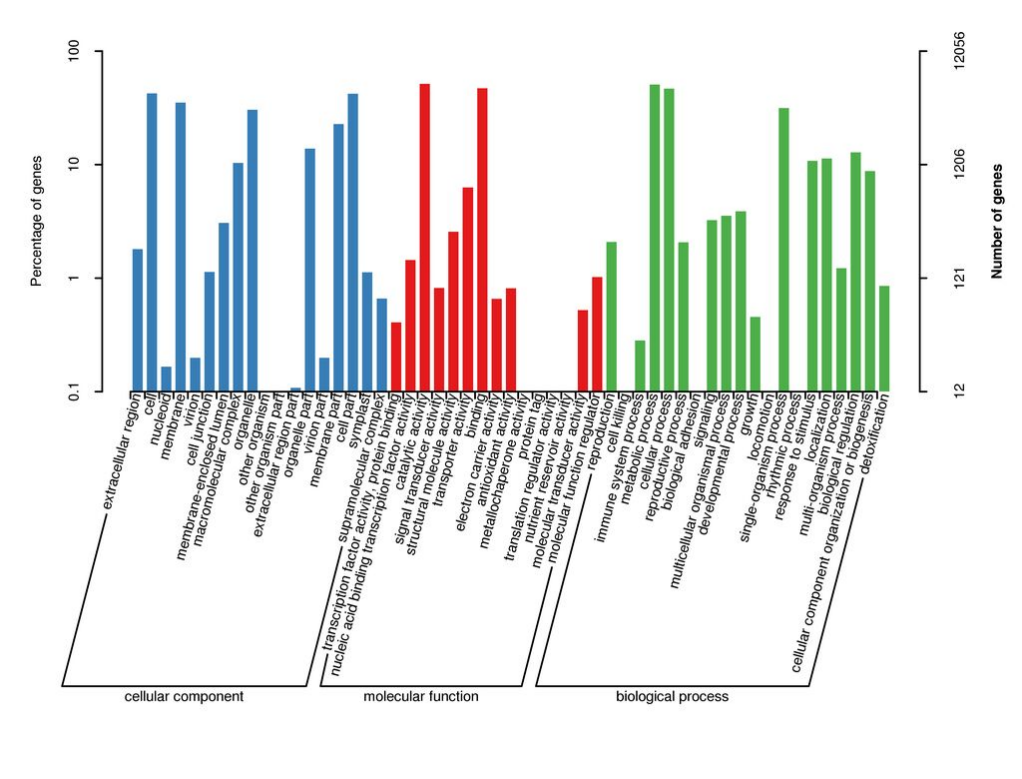
Gene Ontology (GO) classification of unigenes in transcriptome for *C.konishii*.

According to the genes successfully annotated by Nr, GO function classification annotation was performed, and the result is shown in Fig. 2. The analysis results show that 58,905 Unigenes have annotated 12,056 GO functions, accounting for 20.47% of the total number of Unigenes. Divided into three major functional categories, there are 24,768 gene sequences of cellular component functional categories, accounting for 40.35% of the total; 22,969 gene sequences of biological process functional categories, accounting for 37.42% of the total and 13,641 gene sequences of molecular functional categories, accounting for 22.22% of the total. It can be observed that the proportion of genes annotated in the functional category of a cellular component is the largest. The three functional categories can be further divided into 56 GO functional subcategories, including 18 (cellular component), 15 (molecular function) and 20 (biological process). Among the 20 functional subcategories in biological processes: metabolic processes (6,126; 26.67%), cellular processes (5,620; 24.47%) and single-organism processes (93,799; 16.54%) have received more annotations, accounting (5,620; 24.47%), and 16.54% of this type. The percentage of annotations obtained during cell killing in biological processes was the least, having only 0.004%. In the category of cellular components, most of the annotations for cell (5,101; 20.60%), cell part (5,072; 20.50%), membrane (4.253; 17.17%), and organelle (3,659; 14.47%) have been accounted for the other organism (1), another organism part (1), and extracellular region part (13) were less annotated, accounting for 0.004%, 0.004%, and 0.05%, respectively. In the molecular function category, there are many annotations for catalytic activity (6,193) and binding (5682), each accounting for 46.4% and 41.65% of the total classification, while the annotations for translation regulator activity (1), metallochaperone activity (2) and protein tag (3) account for at least 0.007%, 0.015% and 0.022%, respectively. The above GO function annotation results show the basic situation of gene expression in *C.konishii*. It can be seen that among the 3 functional subcategories, there are more genes related to metabolic activities in biological processes, indicating that *C.konishii* has a strong metabolic ability.

**Figure 3.**
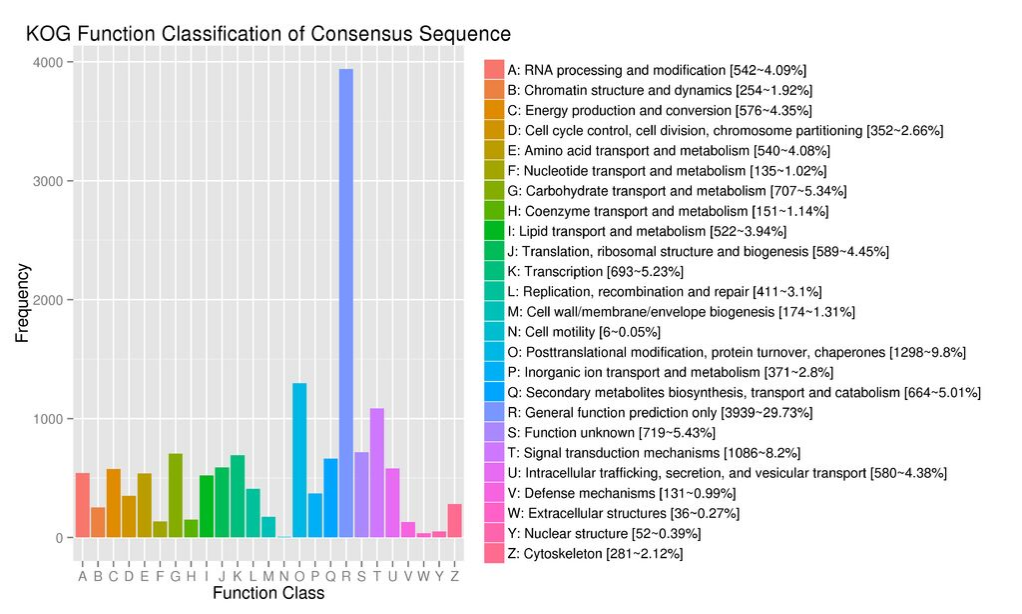
KOG functional annotation distribution of unigenes in transcriptome for *C.konishii*.

The obtained Unigene is classified and annotated by the KOG protein database, and the result is shown in Fig. 3. The analysis results show that 13,248 unigenes in KOG can be matched, accounting for 22.49% of the total. The comparison results can be divided into 25 functional categories, including energy production and conversion, translation, ribosomal structure and biogenesis and different types of gene expression, such as biosynthesis, processing and secondary metabolites biosynthesis, transport and catabolism. Amongst them, there is mainly the general function prediction only (3,939), accounting for 29.73% of the total, followed by post-translational modification, protein turnover and chaperones, accounting for 9.8% of the total; signal transduction mechanisms (8.2%); carbohydrate transport and metabolism (5.34%); and secondary metabolites biosynthesis, transport and catabolism (5.01%). The functional genes for transcription (5.23%) also account for a high proportion. The extracellular structures and cell motility have the least functional annotation information, only five and 36, which account for 0.05% and 0.27% of the total, respectively. C.konishii has more gene expression in terms of transcription, translation and protein transport. In addition, there is one unknown protein whose specific biological function cannot be identified, accounting for 5.43% of the total.

**Figure 4.**
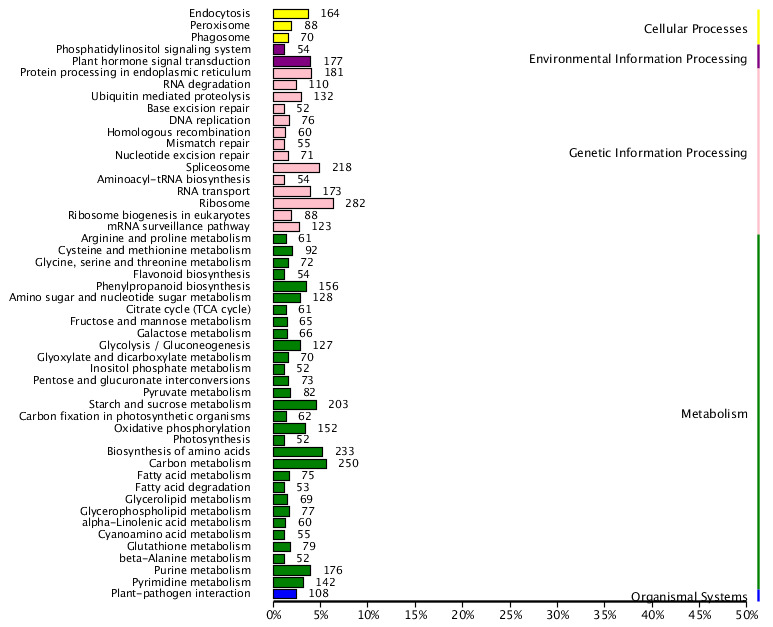
KEGG classification of C.konishii unigene.

Comparing the obtained unigenes to the KEGG database, 8,714 unigenes were annotated, accounting for 14.79% of the total. According to the metabolic pathways involved, C.konishii can be classified into five major categories and 50 subcategories. The results are shown in Fig. 4. Through the specific analysis and statistics of unigenes under the relevant pathway classification in Fig. 4, it was found that the metabolic pathways account for the largest proportion of the five categories, with 2,949 unigenes, followed by pathways related to environmental information processing (1,675 unigenes), genetic information processing (231 unigenes) and cellular processes (322 unigenes); pathways related to organic systems (108) were the fewest. The five major categories were further subdivided into subcategories. Amongst them, metabolism-related pathways were divided into 30 subcategories. Carbon metabolism (250) was the majority, followed by biosynthesis of amino acids (233) and starch and sucrose metabolism (203). The beta-alanine metabolism (52), inositol phosphate metabolism (52) and photosynthesis (52) of subcategories were the least. In addition, pathways related to genetic information processing were divided into 14 subcategories. Ribosome pathways (282) accounted for the most significant number, followed by spliceosome (218) and RNA transport (173). Base excision (52) and aminoacyl-tRNA biosynthesis (52) accounted for the smallest proportion. In the environmental information processing pathways, only two subcategories were included, with plant hormone signal transduction (177) being the majority, followed by the phosphatidylinositol signalling system (54). In the cellular processes, there were three subcategories, including endocytosis (164), peroxisome (88) and phagosome (70). Pathways related to organic systems have only one subcategory (plant-pathogen interaction), accounting for 108 unigenes. In the KEGG metabolic pathway analysis results, the category with the most annotated genes indicated that *C.konishii* has strong metabolic activities (Fig. 4).

### C.konishii essential oil biosynthesis candidate genes

Based on the annotation results, transcripts encoding all the known enzymes involved in essential oil biosynthesis and modification were identified in our dataset (Suppl. material 7). In most cases, multiple unigenes were annotated corresponding to the same enzyme. Such unigenes may represent different fragments of a single transcript or gene family members. This Illumina transcriptome dataset discovered the main identified triterpene saponin biosynthetic genes from C.konishii. These genes include Acetyl-CoA carboxylase (EC:6.4.1.2 6.3.4.14), Enoyl-[acyl-carrier protein] reductase I (EC:1.3.1.9 1.3.1.10), Fatty acyl-ACP thioesterase A (EC:3.1.2.14) and Fatty acyl (SAD; EC:1.14.19.1). It is possible that the discovery of many genes connected to the essential oil pathway will assist us in determining what causes the high essential oil content in C.konishii. The discovery of many genes related to the essential oil pathway may help us to investigate the content of essential oil in *C.konishii*.

### Frequency and distribution of SSRs in the unigenes

All 13,437 assembled unigenes with 27,649,667 bp were examined to discover potential EST-SSRs (Suppl. material 8). A total of 2,854 SSRs were found amongst 58,905 unigenes. Of the 2,854 SSRs, 2,413 sequences contained at least one microsatellite locus, 372 sequences contained more than one microsatellite locus and 98 SSRs involved in compound formation.

The frequency of microsatellites in the *C.konishii* unigenes was 21.24%, with an average of one SSR per 9.688 kb. Of the 2,854 potential SSRs, distribution to different repeat type classes was identified (Suppl. material 4): mononucleotide repeats and trinucleotide repeats were the most abundant, with 1,883 (65.98%) and 652 (22.85%), respectively, followed by dinucleotide repeats (287, 10.06%), tetranucleotide repeats (19, 0.67%), pentanucleotide repeats (3, 0.11%) and hexanucleotide repeats (10, 0.35%). It can be seen that the main repeat types of microsatellite sites in the *C.konishii* transcriptome are mononucleotide repeats, followed by trinucleotide repeats and dinucleotide repeats.

Regarding the number of repeat units, the distributions and frequencies of nucleotide repeats are presented in Table 3. The microsatellites that contained ten repeat units were the most prevalent (744, 26.07%), followed by those that contained five tandem repeats (439, 15.38%), eleven tandem repeats (325, 11.39%) and six tandem repeats (311, 10.90%), while the remaining tandem repeats contributed to less than 10% of the total SSR.

Through statistical analysis of EST-SSR, based on motif types in the *C.konishii* transcriptome, the results are shown in Suppl. material 9. In the mononucleotide repeats, A/T had a large proportion (1,874, 65.66% of all SSRs), followed by C/G (9, 0.32%). In the dinucleotide, the dominant nucleotide repeats were AT/AT (134, 4.7%), followed by AG/CT (117, 4.1%) and AC/GT (36, 1.26%). In the trinucleotide, AGG/CTT was the most common (169, 5.92%), followed by AGC/CTG (122, 4.27%), AGG/CCT (112, 3.92%), ATC/ATG (77, 2.7%), AAT/ATT (54, 1.89%), AAC/GTT (39, 1.37%), CCG/CGG (36, 1.26%), ACC/GGT (30, 1.05%), ACG/CGT (9, 0.32%) and ACT/AGT (4, 0.14%). In the tetranucleotide, AATG/ATTC was the most common (4, 0.14%), followed by AAGG/CCTT (3, 0.11%), AAAT/ATTT (2, 0.07%) and AATC/ATTG (2, 0.07%). Additionally, in the pentanucleotide and hexanucleotide repeats, the types of repeat motifs were evenly distributed, which is related to the small number of corresponding microsatellite sites.

### Genetic diversity

Of the 960 EST-SSR primers developed for *C.konishii*, 99 were able to amplify the target sequences successfully. The sequences of these 99 SSR loci were deposited into NCBI (accession numbers: MW366236 to MW366334). Using 10 different polymorphic EST-SSRs, a study was conducted on the standard genetic diversity and the population genetic structure of three different *C.konishii* populations (Suppl. material 10). Null allele frequencies were determined at four loci for *C.konishii* at the significant level of 0.05. Deviation from HWE, due to excessive heterozygosity, was significant at four loci of MP16, MP17, MP21 and MP28 in *C.konishii* (Table 4). Forty-six alleles across 96 *C.konishii* plants were produced from the ten polymorphic microsatellite markers (Table 4). Table 4 shows the values: Na = 4.03 (2.67-6.67), Ne = 2.76 (1.25-5.23), PIC = 0.67 (0.52-0.82), Ho = 0.56 (0.19-078) and He = 0.58 (0.2-0.96). The fixation index (F_IS_) was 0.03. Positive F_IS_ values were found across all five loci, indicating an excessive number of homozygotes and suggesting inbreeding. Nonetheless, these loci exhibited high inbreeding (*p* < 0.05). It may be deduced that the inbreeding coefficient of the populations (F_IT_ = 0.06) suggests an excess of homozygosity in the populations (Table 5).

The number of polymorphic loci varied between populations for *C.konishii*. The percentage of polymorphic loci was high (100%) in all *C.konishii* populations (Table 5). The average alleles per locus (N_a_) was 4.03, ranging from 4.0 in XL and HSP to 4.1 in the PH population for *C.konishii*. The highest private alleles (N_P_) level was observed in XL (N_P_ = 0.4). The lowest value of effective alleles (Ne) was observed in the XL population (Ne = 2.39), whereas this value was the highest in the PH population (Ne = 3.03, Ho = 0.56 and He = 0.58). The lowest values of Ho = 0.50 and He = 0.55 were detected in the XL population. The F_IS_ was 0.06 (0.02-0.1). Significantly positive F_IS_ values were observed in the XL population, showing a deficiency of heterozygotes. *F*_IS_IIM = 0.019 showed the F_IS_IIM values varied from 0.14 (PH) to 0.025 (HSP), with an average of 0.019. Both the PH and the HSP populations of the *C.konishii* species experienced a recent bottleneck in their populations (Table 5).

### Genetic differentiation and genetic structure

The analysis of molecular variance (AMOVA) was implemented, based on 1023 permutations and revealed that the molecular variation attributable to differentiation amongst and within the populations for *C.konishii* (Table 6). The fixation index (F_ST_), which can measure genetic differentiation amongst loci and averaged value was 0.03 (0.00-0.06) for *C.konishii* (Table 4), showing that 2.97% and 6.35% of the genetic variation existed between populations for *C.konishii* (Table 6). The F_ST_ value amongst populations varied from 0.014 (HSP and PH) to 0.082 (XL and PH). Significant differentiation was observed for pairwise F_ST_ values for both the *C.konishii* species (p < 0.05 and 0.01), except for the pair of HSP and PH (Suppl. material 11). Based on the Fst values matrix, the NJ analysis showed the two main groups (Fig. 6). The first group included the population of HSP in the northeast area. The second group included the two populations of XL and PH in the north-central area, with a bootstrap of 100%. The Mantel test based on genetic distance and geographical distance amongst populations is shown in Suppl. material 5. Mantel analysis found significant differences in mean scores on the Nei’s genetic distance and geographic distance matrix amongst populations (R^2^ = 0.8492).

**Figure 5.**
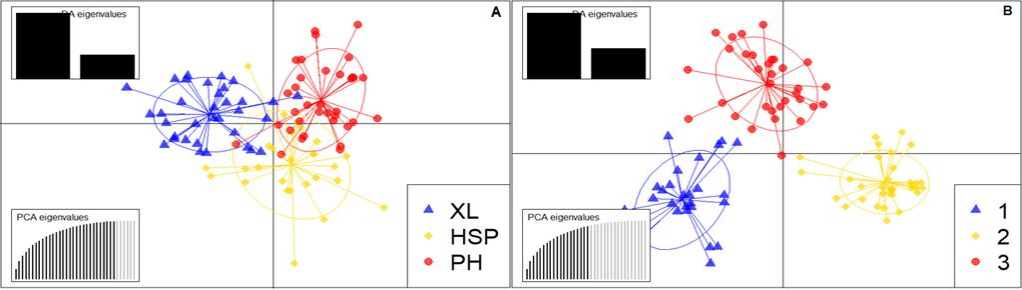
Analysis of population structure using DAPC. Scatterplot of the DAPC with prior information (A) and Scatterplot of the DAPC without prior information (B) for *C.konishii*.

**Figure 6.**
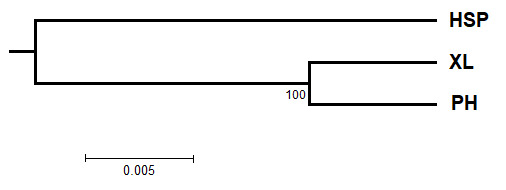
Genetic relationships amongst populations, based on Neighbour – Joining (NJ).

Without any prior information, discriminant analysis of principal components (DAPC) also uncovered three genetic groupings for *C.konishii* (Fig. 5). Cluster 1 included 12 individuals from the XL population, seven individuals from the HSP population and 10 individuals from the PH population (Fig. 1, Suppl. material 12). Most individuals from the two populations of PH (16 individuals) and HSP (11 individuals) were assigned to cluster 2. In cluster 2, there were also seven individuals in XL. Cluster 3 included most individuals in XL and nine in HSP and PH.

**Figure 7.**
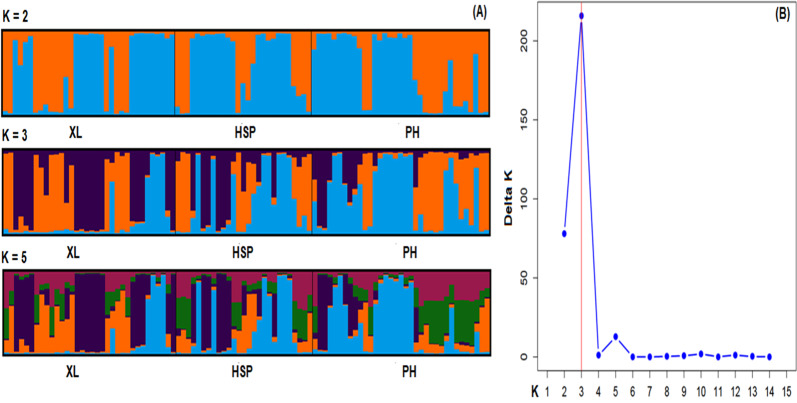
Bar plots for three *C.konishii* populations at K = 2, K = 3 and K = 5 (A) and Mean values of distribution probability of the delta K with standard deviation from 10 runs for each value of K = 1–15 (B).

The DAPC, with prior information on population origin, showed individuals within and between populations (Fig. 5). The high overlap indicated low genetic differentiation between populations. Our findings showed high overlaps between XL and HSP, with an F_ST_ value of 0.034. Similarly, low overlaps were found between HSP and PH, with an F_ST_ value of 0.014. The Bayesian analysis of the assignation of individuals, based on the likelihoods, showed that the highest ∆K value (215.9) for 96 individuals revealed K = 3 to be an optimum number of genetic groups and showed that all individuals exhibited admixture from three groups (Figs 1, 7). The proportion of ancestry linked with each genetic group might be deduced from the colour of each individual, as measured by their percentage of the population's total colour (Fig. 7). One group (orange) was predominant in the XL populations with strong ancestry values of 47.3% and two populations (HSP and PH) had lower ancestry values of 29.4% and 16%, respectively (Suppl. material 13). In the second group (blue), two populations (PH and HSP) represented the largest group (47.7% and 37.9%, respectively), followed by the XL population (16%). Similarly, for the last group (purple), the three populations (XL, HSP and PH) were the same (36.8%, 32.7% and 36.3%, respectively). At K = 2, the *C.konishii* populations were divided into two groups. At K = 5, the central-southeast group was diﬀerentiated into five groups.

## Discussion

The Illumina HiSeq4000 sequencing platform was used in this study to determine the transcriptome of *C.konishii* and bioinformatics techniques were used to analyse the microsatellite sites in the transcriptome database. The transcriptome sequencing of *C.konishii* provided a valuable resource for developing SSR markers to study genetic diversity, molecular marker-assisted breeding and the evolution of the Cupressaceae family. Fifty-eight thousand nine hundred and five (58905) transcriptomic unigenes were obtained using the Illumina HiSeq4000 platform for *C.konishii*, with a mean length of 763.4 bp and N50 of 1,447 bp. These lengths are shorter than the other conifer species, such as *Torreyagrandis* (Taxaceae) with N50 = 1,503 bp and average length = 766 bp (Zeng et al. 2018), *Cunninghamialanceolata* (Cupressaceae) with N50 = 1,566 (Lin et al. 2020), but longer than *Amentotaxusargotaenia* (Taxaceae) with N50 = 947 bp, mean length = 721 bp (Ruan et al. 2019), indicating that the transcriptome sequencing data were well assembled for *C.konishii* in the present study. The methods used in the current study are the same. However, the deviation might be due to the sequencing depth, assembly method and natural characteristics of the species. After assembling the uni-transcripts, the GC content (44.45%) of *C.konishii* was lower than that of *Amentotaxusargotaenia* (GC = 47%) (Ruan et al. 2019), but higher than those of *Torreyagrandis* (GC = 43.54%) (Zeng et al. 2018) and *Cunninghamialanceolata* (GC = 36.04%) (Lin et al. 2020) although the methodology was the same. The GC content provides information regarding the stability of genes and genomic composition for the evolution and genetic structure of the species. These results can be related to species' adaptability to different environmental conditions. The COG, GO, KEGG, KOG, Pfam, Swiss-Prot and NR databases were searched for sequence matches in order to determine the genetic diversity of *C.konishii* and its development. Annotations of GO categories were made on the *C.konishii* unigenes. The metabolic process in the biological categories and cells in cellular components were the largest groups in this study, indicating important cellular and metabolic activities. These findings are comparable to those found in an earlier investigation of *Torreyagrandis* (Zeng et al. 2018), *Amentotaxusargotaenia* (Ruan et al. 2019) and *Cunninghamialanceolata* (Lin et al. 2020). Thirteen thousand two hundred and forty eight (13,248) unigenes were assigned to 25 KOG categories of *C.konishii*. The current KOG database results are parallel to those previously reported for 26 in KOG of *Torreyagrandis* (Zeng et al. 2018) and 25 in KOG of *Amentotaxusargotaenia* (Ruan et al. 2019). For this purpose, five major categories and 50 subcategories in KEGG resources were used to study genes' biological functions and interactions. Some dominant pathways were ribosome, oxidative phosphorylation, protein processing in the endoplasmic reticulum, glycolysis/gluconeogenesis, spliceosome, RNA transport and purine metabolism. These findings suggest that the *C.konishii* unigenes discovered in the study have broad applicability and will be valuable in the future studies of the species' functional diversity.

The average length of the unigenes found in *C.konishii* was significantly longer than that of other conifers, such as *A.argotaenia* (721 bp), *Pinuspinaster* (495 bp) (Canales et al. 2013), *Platycladusorientalis* (686 bp) (Hu et al. 2016) and *P.abies* (472 bp) (Chen et al. 2012). According to Zalapa et al. (2011), having longer sequences will enhance the likelihood of successfully developing EST-SSR primers, so *C.konishii* is chosen as a prime target for microsatellite analysis in this study. Furthermore, the results indicate 58,905 unigenes in the transcriptome of *C.konishii*. There are 2,854 microsatellite loci and the frequency of occurrence is 21.24%. At the same time, there are abundant types of microsatellites in the transcriptome of *C.konishii*, with six different types of nucleotide repeats, of which the main repeat type is mononucleotide repeats, accounting for 65.98% of the total number of microsatellite loci; trinucleotide repeats, accounting for 22.85% of the total number of microsatellite loci, which are similar to *Picea* spp. (Rungis et al. 2004), *Pinusdabeshanensis* (Xiang et al. 2015), *Pseudolarixamabilis* (Geng et al. 2015), *Torreyagrandis* (Zeng et al. 2018), *Amentotaxusargotaenia* (Ruan et al. 2019) and *C.lanceolata* (Lin et al. 2020). According to Ranade et al. (2014), the AT/TA dimer motif is the most common in gymnosperms, particularly in 3' untranslated regions. Rising levels of A+T might have a role in this occurrence. As shown in Suppl. material 9, the most common trinucleotide pattern found in *C.konishii* was AAG/CTT, which is comparable to that found in *Cryptomeriajaponica* (Ueno et al. 2012), *Pinustaeda* (Wegrzyn et al. 2014) and *P.halepensis* (Pinosio et al. 2014), but different from that found in *P.dabeshanensis* (Xiang et al. 2015). The AAG/CTT complex has been determined to be the methylation target in plants (Law and Jacobsen 2010).

Improvements to germplasm resources through genetic diversity have been extensively implemented in several other conifers (Zeng et al. 2018, Ruan et al. 2019, Lin et al. 2020). Generally, the level of genetic diversity within species correlates with number of loci and population, the size of its geographical range (Nguyen et al. 2020) and the extent of genetic exchange (Hellmann and Pineda-Krch 2007). According to the evaluation criteria of polymorphism (PIC > 0.5) (Botstein et al. 1980), the current study found evidence of high genetic variation, based on the EST-SSR (PIC = 0.67). Due to the relatively high PIC values, the chosen loci are both highly informative and well suited for genetic research on *C.konishii*. Several genetic measurements, including the mean number of observed alleles, observed heterozygosity, expected heterozygosity and the percentage of the polymorphic band, corroborate this finding (Lin et al. 2020). *C.konishii* exhibited a moderate degree of genetic diversity (He = 0.58; Ho = 0.56) compared to other conifers, such as *C.lanceolata* (He = 0.573; H_O_ = 0.526) (Lin et al. 2020), *Torreyagrandis* (Ho = 0.54) (Zeng et al. 2018) and *Taxus ﬂorinii* (Ho = 0.578; He = 0.526) (Qin et al. 2017). Furthermore, our results showed that the observed number of alleles for each locus in *C.konishii* (Na = 4.03) was lower than that in *C.lanceolata* (Na =6.47) (Lin et al. 2020). The extensive genetic diversity within a species is a reflection of its evolutionary past and the ecogeographic environment in which it has thrived. It is common for species to maintain high genetic diversity when they exhibit a wide distribution pattern, a large population size, a long lifespan, a predominance of outcrossing and successional stages (Hamrick and Godt 1996, White et al. 2007). It is hypothesised that species with high genetic heterozygosity will have a greater capacity for adaptability in their environment. This hypothesis is supported by the observation that conifers have a broad distribution and exceptional longevity. However, similar results in the present study have been reported in previous studies, such as the sister species of *C.konishii*, Chinese fir - *Cunninghamialanceolata* with Ho = 0.56 and He = 0.604 (Duan et al. 2017) and *Pinuskoraiensis* with Ho = 0.299 and He = 0.311) (Li et al. 2020). Genetic heterozygosity is related to anthropogenic disturbance and can be reduced through genetic drift and increased homozygosity for common alleles due to the loss of rare alleles (Falk and Holsinger 2023). The moderate genetic heterozygosity in XL, HSP and PH suggests potential antrophogenic influence. In all studied populations, deforestation and overexploitation are key factors reducing genetic heterozygosity. Most of the conifer trees with more than 60 cm diameter were exploited by forestry enterprises in the 1980s and 1990s (Most and Vast 2024). The recent populations include trees less than 100 years old that have fragmented into small populations. The short evolutionary history and tiny population sizes of *C.konishii* may account for its moderate genetic diversity. An effective population size and genetic variety decrease due to inbreeding may have resulted from anthropogenic disturbance and population declines. Significant heterozygosity deficits across 96 trees from three populations suggest a relatively high heterozygosity deficiency can exist within the *C.konishii* populations. This shows that inbreeding exists in small populations, although this species is outcrossed (Murawski et al. 1994). Bottleneck-determined heterozygosity deficits and bottleneck events were identified in the PH population.

In the present study, the genetic differentiation amongst *C.konishii* populations was detected by F_ST_ = 0.029, consistent with the AMOVA analysis results. The genetic differentiation of *C.konishii* was similar to that of *C.konishii* in China using AFLP markers (F_ST_ = 0.048, P = 0.016) (Chung et al. 2004). Gene flow and random mutations contribute to genetic diversity amongst populations (Schaal et al. 1998). Low population genetic differentiation reflects high gene flow. Strong gene flow (Nm > 1) determined a high number of migrants for each generation and may prevent genetic differentiation amongst populations due to genetic drift (Slatkin 1987). According to our study, a gene flow rate was comparatively high (Nm = 8.169) could mitigate the genetic drift effects by lowering the population's genetic differentiation, while increasing genetic heterozygosity. Gene flow is determined via the dispersal of pollen grains and seeds (Finkeldey and Hattemer 2007) and contributes to population genetic differences and genetic structure. Conifers are long-lived, predominantly outcrossed, insect-pollinated and late successional (Murawski et al. 1994). Their seeds are dispersed by wind and water (Ashton 1982). Insects are responsible for pollinating *C.konishii*, while the wind is responsible for spreading its seeds. The increased isolation distance will result in a reduction in the amount of pollen that is transmitted from population to population of *C.konishii*. Pollen transmission is primarily associated with insects. The current population is fragmented into smaller populations. Thus, habitat fragmentation leading to increased isolation can decrease gene exchange, which can influence its genetic structure in recent decades. The genetic structure of *C.konishii* in the present study was also detected via different clustering analyses (Structure and DAPC), in which 96 studied trees were grouped, based on their geographic origin. Therefore, the genetic structure's existence could result from gene exchange related to geographic distance and habitat fragmentation that separates populations into different genetic groups. Thus, a decrease in the dispersal of pollen and seeds might restrict gene flow for *C.konishii*.

## Conclusions

The *C.konishii* transcriptome was sequenced for the first time using the Illumina 4000 sequencing technology. Many ESTs were generated and differentially expressed genes in *C.konishii* were found. A total of 2854 EST-SSRs were discovered and 99 microsatellite sites were successfully deposited into GenBank (accession numbers: MW366236 to MW366334). It is clear from the results that the naturally occurring populations of *C.konishii* have kept a reasonable amount of genetic variation over time. Several SSR markers were found, all of which will be useful in the future marker-assisted breeding of *C.konishii*. We determined a moderate genetic variation and low genetic difference amongst the three populations of *C.konishii*. Without prior information, the structure and the DAPC identified three main genetic groups besides moderate genetic diversity in three populations of conifer species. Thus, three populations might be prioritised for in situ conservation. Simultaneously, seeds from these populations should be collected for ex-situ conservation activities. An increase in population sizes can prevent a reduction in genetic diversity via genetic drift, homozygosity for common alleles and limited gene migration across populations. This research lays the foundation for future essential oil extraction from *C.konishii* plants and conservation efforts to protect the species' genetic diversity.

## Supplementary Material

DBF466DF-2F26-52A7-A059-043C7E2B7CDA 10.3897/BDJ.13.e153663.suppl1 Supplementary material 1 Length distribution of assembly transcript Data type images Brief description Length distribution of assembly transcript for *C.konishii*. File: oo_1376047.png https://binary.pensoft.net/file/1376047 Bich Hong Ha, Mai Phuong Pham, Quoc Khanh Nguyen, Thi Tuyet Xuan Bui, Dinh Giap Vu, Syed Noor Muhammad Shah, Dinh Duy Vu

4490C055-C705-5459-86C5-1762F2D671B3 10.3897/BDJ.13.e153663.suppl2 Supplementary material 2 Length distribution of assembly unigenes Data type Images Brief description Length distribution of assembly unigenes for *C.konishii*. File: oo_1278577.png https://binary.pensoft.net/file/1278577 Bich Hong Ha, Mai Phuong Pham, Quoc Khanh Nguyen, Thi Tuyet Xuan Bui, Dinh Giap Vu, Syed Noor Muhammad Shah, Dinh Duy Vu

96AED643-6CBA-57D3-9CF0-E0C3DE151F3D 10.3897/BDJ.13.e153663.suppl3 Supplementary material 3 Distribution of species search of unigenes Data type images Brief description Distribution of species search of unigenes against the Nr database of *C.konishii*. File: oo_1278579.jpg https://binary.pensoft.net/file/1278579 Bich Hong Ha, Mai Phuong Pham, Quoc Khanh Nguyen, Thi Tuyet Xuan Bui, Dinh Giap Vu, Syed Noor Muhammad Shah, Dinh Duy Vu

AB9570A1-30B5-5FDA-AE1F-3850B946AC63 10.3897/BDJ.13.e153663.suppl4 Supplementary material 4 Distribution of different repeat type classes Data type images Brief description Distribution of different repeat type classes in *C.konishii* transcriptome. File: oo_1278598.png https://binary.pensoft.net/file/1278598 Bich Hong Ha, Mai Phuong Pham, Quoc Khanh Nguyen, Thi Tuyet Xuan Bui, Dinh Giap Vu, Syed Noor Muhammad Shah, Dinh Duy Vu

DB360A2F-F7AB-5310-A542-31D8638284EF 10.3897/BDJ.13.e153663.suppl5 Supplementary material 5 Mantel test of genetic distance and geographical distance Data type images Brief description Mantel test of genetic distance and geographical distance of three *C.konishii* populations. File: oo_1278599.png https://binary.pensoft.net/file/1278599 Bich Hong Ha, Mai Phuong Pham, Quoc Khanh Nguyen, Thi Tuyet Xuan Bui, Dinh Giap Vu, Syed Noor Muhammad Shah, Dinh Duy Vu

86B73687-3E61-5F88-AB67-646AD1E44180 10.3897/BDJ.13.e153663.suppl6 Supplementary material 6 Table S1. Sampling location Data type Table S1 Brief description Table S1. Sampling location for *C.konishii* from Vietnam in the present study. File: oo_1278605.docx https://binary.pensoft.net/file/1278605 Bich Hong Ha, Mai Phuong Pham, Quoc Khanh Nguyen, Thi Tuyet Xuan Bui, Dinh Giap Vu, Syed Noor Muhammad Shah, Dinh Duy Vu

95C49F70-4008-5360-9557-69FF8A3FF699 10.3897/BDJ.13.e153663.suppl7 Supplementary material 7 Table S2. The main identified essential oil biosynthetic genes Data type Table S2 Brief description Table S2. The main identified essential oil biosynthetic genes from *C.konishii* unigenes. File: oo_1278609.docx https://binary.pensoft.net/file/1278609 Bich Hong Ha, Mai Phuong Pham, Quoc Khanh Nguyen, Thi Tuyet Xuan Bui, Dinh Giap Vu, Syed Noor Muhammad Shah, Dinh Duy Vu

35530073-A3C8-5F9E-9716-89BB916EF474 10.3897/BDJ.13.e153663.suppl8 Supplementary material 8 Table S3. Summary of analyses of expressed sequence Data type Table S3 Brief description Table S3. Summary of analyses of expressed sequence Tag–Simple Sequence repeat (EST-SSRs) in *C.konishii*. File: oo_1278611.docx https://binary.pensoft.net/file/1278611 Bich Hong Ha, Mai Phuong Pham, Quoc Khanh Nguyen, Thi Tuyet Xuan Bui, Dinh Giap Vu, Syed Noor Muhammad Shah, Dinh Duy Vu

FBC17932-AE3F-5526-A33B-1F55D668A263 10.3897/BDJ.13.e153663.suppl9 Supplementary material 9 Table S4. Frequency distribution of SSRs Data type Table S4 Brief description Frequency distribution of SSRs, based on motif types in *C.konishii* transcriptome. File: oo_1278613.docx https://binary.pensoft.net/file/1278613 Bich Hong Ha, Mai Phuong Pham, Quoc Khanh Nguyen, Thi Tuyet Xuan Bui, Dinh Giap Vu, Syed Noor Muhammad Shah, Dinh Duy Vu

1C618873-B161-57B0-9BC8-E4DDC83D96AD 10.3897/BDJ.13.e153663.suppl10 Supplementary material 10 Table S5. Primer sequences, repeat motif, size range of alleles and annealing temperature Data type Table S5 Brief description Table S5. Primer sequences, repeat motif, size range of alleles and annealing temperature (Tm) of 10 polymorphic EST-SSR markers developed for *C.konishii*. File: oo_1278615.docx https://binary.pensoft.net/file/1278615 Bich Hong Ha, Mai Phuong Pham, Quoc Khanh Nguyen, Thi Tuyet Xuan Bui, Dinh Giap Vu, Syed Noor Muhammad Shah, Dinh Duy Vu

117716DD-A238-5745-A494-830EB7D485D3 10.3897/BDJ.13.e153663.suppl11 Supplementary material 11 Table S6. Pairwise genetic differentiation (F_ST_) between populations Data type Table S6 Brief description Table S6. Pairwise genetic differentiation (F_ST_) between populations for *C.konishii* species. File: oo_1278616.docx https://binary.pensoft.net/file/1278616 Bich Hong Ha, Mai Phuong Pham, Quoc Khanh Nguyen, Thi Tuyet Xuan Bui, Dinh Giap Vu, Syed Noor Muhammad Shah, Dinh Duy Vu

E4D661B0-7413-53FC-B2B9-DB4E013AA935 10.3897/BDJ.13.e153663.suppl12 Supplementary material 12 Table S7. Number of individuals for each population assigned Data type Table S7 Brief description Table S7. Number of individuals for each population assigned. Each cluster was obtained from DAPC without prior information. File: oo_1278617.docx https://binary.pensoft.net/file/1278617 Bich Hong Ha, Mai Phuong Pham, Quoc Khanh Nguyen, Thi Tuyet Xuan Bui, Dinh Giap Vu, Syed Noor Muhammad Shah, Dinh Duy Vu

D23BFBCD-4807-5C35-A866-C4DB4ED1AE73 10.3897/BDJ.13.e153663.suppl13 Supplementary material 13 Table S8. Percentage of ancestry Data type Table S8 Brief description Table S8. Percentage of ancestry for three *C.konishii* populations. File: oo_1278619.docx https://binary.pensoft.net/file/1278619 Bich Hong Ha, Mai Phuong Pham, Quoc Khanh Nguyen, Thi Tuyet Xuan Bui, Dinh Giap Vu, Syed Noor Muhammad Shah, Dinh Duy Vu

## Figures and Tables

**Table 1.** Length distribution of assembly transcript and unigenes for *C.konishii*.

**Length range (bp)**	**Unigene**	**Transcripts**
200-300	21,752 (36.93%)	25,236 (25.31%)
300-500	14,338 (24.34%)	18,932 (19.06%)
500-1000	9,378 (15.92%)	18,346 (18.47%)
1000-2000	7,987 (13.56%)	20,870 (21.01%)
> 2000	5.450 (9.25%)	16,041 (16.15%)
Total Number	58,905	99,325
Total Length	44,965,632	105,702,940
N50 Length	1,447	1,869
Mean Length	763.4	1,064

**Table 2.** Functional annotation of *C.konishii* in different databases.

**Annotated database**	**Annotated_No.**	**Percentage (%)**	**300–1000 (bp)**	≥ **1000 (bp)**
**COG**	7,892	13.40	1,591	5,357
**GO**	12,056	20.47	3,724	6,233
**KEGG**	8,714	14.79	2,411	5,244
**KOG**	13,248	22.49	3,818	7,438
**Pfam**	16,688	28.33	4,458	10,297
**Swissprot**	16,510	28.03	5,133	9,073
**eggNOG**	21,467	36.44	6,487	11,728
**Nr**	23,232	39.44	7,371	12,085
**All**	23,878	40.54	7,597	12,128

**Table 3.** Frequency of SSRs, based on repeat types in *C.konishii* transcriptome.

**Number of repeats**	**Repeat type**						**Total**	**Percentage (%)**
	**Mono**-	**Di**-	**Tri**-	**Tetra**-	**Penta**-	**Hexa**-		
**5**	-	-	416	13	3	7	439	15.38
**6**	-	147	155	6		3	311	10.90
**7**	-	50	71				121	4.24
**8**	-	28	9				37	1.30
**9**	-	19					19	0.67
**10**	728	15	1				744	26.07
**11**	298	27					325	11.39
**12**	165	1					166	5.82
**13**	106						106	3.71
**14**	70						70	2.45
**15**	61						61	2.14
**16**	55						55	1.93
**17**	50						50	1.75
**18**	73						73	2.56
**19**	85						85	2.98
**20**	106						106	3.71
**21**	62						62	2.17
**22**	18						18	0.63
**23**	6						6	0.21
**Total**	1883	287	652	19	3	10	2854	
**Percentage (%)**	65.98	10.06	22.85	0.67	0.11	0.35		

**Table 4.** Genetic parameters in ten SSR loci for *C.konishii*.

**Locus**	***N_a_***	***N_e_***	**Null allele**	**PIC**	***H*_O_**	***H*_E_**	***F*_IS_**	***F*_IT_**	***F*_ST_**	***P*_HWE_**
**MP01**	5.33	2.74	No	0.76	0.62	0.62	0.01	0.04	0.03	***
**MP 11**	4.00	3.16	No	0.65	0.68	0.88	-0.30	-0.28	0.01	ns
**MP 13**	3.33	2.58	No	0.70	0.59	0.83	-0.39	-0.31	0.06	***
**MP 15**	4.00	2.81	No	0.64	0.64	0.96	-0.49	-0.49	0.00	***
**MP 16**	3.33	2.48	0.24	0.66	0.60	0.23	0.61	0.62	0.02	***
**MP 17**	4.00	2.05	0.28	0.62	0.51	0.35	0.30	0.31	0.01	**
**MP 18**	3.00	2.40	No	0.52	0.58	0.77	-0.33	-0.29	0.03	**
**MP 21**	6.67	5.23	0.36	0.61	0.78	0.43	0.45	0.47	0.04	***
**MP 28**	4.00	2.90	0.37	0.82	0.65	0.31	0.53	0.56	0.06	***
**MP 30**	2.67	1.25	No	0.70	0.19	0.20	-0.05	0.01	0.05	***
**Mean**	4.03	2.76		0.67	0.56	0.58	0.03	0.06	0.03	
*Note: N_a_* number of alleles, *Ne* effective alleles, PIC polymorphism information content, *H*_O_ and *H*_E_ observed and expected heterozygosity, *F*_IS_, fixation index, Null allele the average null allele frequency, *F*_IT_ coefficient of total inbreeding, *F*_ST_, genetic differentiation index of Weir and Cockerham (1984), ns = not significant, *P < 0.05, **P < 0.01, ***P < 0.001 .										

**Table 5.** Population statistics for different geographical populations of *C.konishii* investigated using ten microsatellite loci.

***Populations***	***P(%)***	***N*_a_**	***N*_e_**	***Np***	***H*_O_**	***H*_E_**	***F*_IS_**	***F*_IS_ IIM**	**P value of bottleneck**		
									**IAM**	**SMM**	**TPM**
**XL**	100	4.00	2.39	0.4	0.50	0.55	0.10^*^	0.018	0.016	ns	ns
**HSP**	100	4.00	2.86	0.1	0.59	0.59	0.02	0.025	0.003	ns	0.012
**PH**	100	4.10	3.03	0.1	0.58	0.61	0.06	0.014	0.001	0.006	0.001
**Mean**	100	4.03	2.76		0.56	0.58	0.06	0.019			
*Note: N*a, mean number of alleles per locus; *N*e, mean number of effective alleles; *Np*, No. Private Alleles. P%, percentage of polymorphic loci; *H*_O_ and *H*_E_, mean observed and expected heterozygosities, respectively; *F*_IS_, inbreeding coefficient with *p < 0.05. **P < 0.01, ***P < 0.001; ns, not significant; The individual inbreeding model was performed to evaluate the *F*_IS_ index for null allele frequency *F*_IS_IIM; IAM, the infinite allele model; SMM, stepwise mutation model; TPM (two-phased model of mutation); XL, Xuan Lien Nature Reserve; HSP, Hoang Su Phi Nature Reserve; PH, Pu Hoat Nature Reserve.											

**Table 6.** Analysis of molecular variance from natural populations for *C.konishii* species produced.

	**df**	**Sum of squares**	**Variance components**	**Total** **variation (%)**	**Fixation indices**
Amongst populations	2	17.894	0.091	2.97	F_IS_ = 0.065** F_ST_ = 0.029*** F_IT_ = 0.093***
Amongst individuals within populations	93	294.336	0.194	6.35	
Within individuals	96	266.500	2.78	90.68	
Total	191	578.729	3.061	100	
*Note*: df, degree of freedom; **P < 0.01, ***p < 0.001					

## References

[B12651523] Apweiler R. (2004). UniProt: the Universal Protein knowledgebase. Nucleic Acids Research.

[B12651543] Ashburner M, Ball C A, Blake J A, Botstein D, Butler H, Cherry J M, Davis A P, Dolinski K, Dwight S S, Eppig J T, Harris M A, Hill D P, Issel-Tarver L, Kasarskis A, Lewis S, Matese J C, Richardson J E, Ringwald M, Rubin G M, Sherlock G (2000). Gene ontology: tool for the unification of biology. The Gene Ontology Consortium.. Nature genetics.

[B12678958] Ashton P. S., (1982). Dipterocarpaceae. In: Steenis, Ed., Malesiana Series I.

[B12651581] Averyanov Leonid V., Nguyen Tien Hiep, Sinh Khang Nguyen, Pham The Van, Lamxay Vichith, Bounphanmy Somchanh, Lorphengsy Shengvilai, Phan Loc Ke, Lanorsavanh Soulivanh, Chantthavongsa Khamfa (2014). Gymnosperms of Laos. Nordic Journal of Botany.

[B12651605] Botstein D, White R L, Skolnick M, Davis R W (1980). Construction of a genetic linkage map in man using restriction fragment length polymorphisms.. American journal of human genetics.

[B12678659] Canales Javier, Bautista Rocio, Label Philippe, Gómez‐Maldonado Josefa, Lesur Isabelle, Fernández‐Pozo Noe, Rueda‐López Marina, Guerrero‐Fernández Dario, Castro‐Rodríguez Vanessa, Benzekri Hicham, Cañas Rafael A., Guevara María‐Angeles, Rodrigues Andreia, Seoane Pedro, Teyssier Caroline, Morel Alexandre, Ehrenmann François, Le Provost Grégoire, Lalanne Céline, Noirot Céline, Klopp Christophe, Reymond Isabelle, García‐Gutiérrez Angel, Trontin Jean‐François, Lelu‐Walter Marie‐Anne, Miguel Celia, Cervera María Teresa, Cantón Francisco R., Plomion Christophe, Harvengt Luc, Avila Concepción, Gonzalo Claros M., Cánovas Francisco M. (2013). De novo assembly of maritime pine transcriptome: implications for forest breeding and biotechnology. Plant Biotechnology Journal.

[B12653590] Chabane K., Ablett G. A., Cordeiro G. M., Valkoun J., Henry R. J. (2005). EST versus genomic derived microsatellite markers for genotyping wild and cultivated barley. Genetic Resources and Crop Evolution.

[B12678711] Chen Jun, Källman Thomas, Ma Xiaofei, Gyllenstrand Niclas, Zaina Giusi, Morgante Michele, Bousquet Jean, Eckert Andrew, Wegrzyn Jill, Neale David, Lagercrantz Ulf, Lascoux Martin (2012). Disentangling the roles of history and local selection in shaping clinal variation of allele frequencies and gene expression in norway spruce *Piceaabies*). Genetics.

[B12651662] Chung J. D., Lin T. P., Tan Y. C., Lin M. Y., Hwang S. Y. (2004). Genetic diversity and biogeography of *Cunninghamiakonishii* (Cupressaceae), an island species in Taiwan: a comparison with *Cunninghamialanceolata*, a mainland species in China. Molecular Phylogenetics and Evolution.

[B12678585] Chybicki Igor J., Burczyk Jaroslaw (2008). Simultaneous estimation of null alleles and inbreeding coefficients. Journal of Heredity.

[B12651672] Conesa Ana, Götz Stefan, García-Gómez Juan Miguel, Terol Javier, Talón Manuel, Robles Montserrat (2005). Blast2GO: a universal tool for annotation, visualization and analysis in functional genomics research. Bioinformatics.

[B12651737] Deng Y. Y.,, Li J. Q.,, Wu S. F.,, Zhu Y. P.,, Cai Y. W., He F. C. (2006). Integrated nr database in protein annotation system and its localization. Computer Engineering.

[B12651772] Ding Mingyan, Meng Kaikai, Fan Qiang, Tan Weizheng, Liao Wenbo, Chen Sufang (2017). Development and validation of EST‐SSR markers for *Fokieniahodginsii* (Cupressaceae). Applications in Plant Sciences.

[B12651801] Duan Hongjing, Cao Sen, Zheng Huiquan, Hu Dehuo, Lin Jun, Cui Binbin, Lin Huazhong, Hu Ruiyang, Wu Bo, Sun Yuhan, Li Yun (2017). Genetic characterization of chinese fir from six provinces in southern China and construction of a core collection.. Scientific reports.

[B12651792] Du Jia, Zhang Zhen, Zhang Hanguo, Junhong Tang (2017). EST–SSR marker development and transcriptome sequencing analysis of different tissues of Korean pine (*Pinuskoraiensis* Sieb. et Zucc.). Biotechnology & Biotechnological Equipment.

[B12651830] Earl Dent A., vonHoldt Bridgett M. (2011). Structure harvester: a website and program for visualizing structure output and implementing the evanno method. Conservation Genetics Resources.

[B12651839] Egan Ashley N., Schlueter Jessica, Spooner David M. (2012). Applications of next‐generation sequencing in plant biology. American Journal of Botany.

[B12653495] Ellis J R, Burke J M (2007). EST-SSRs as a resource for population genetic analyses. Heredity.

[B12653580] Eujayl I., Sorrells M. E., Baum M., Wolters P., Powell W. (2002). Isolation of EST-derived microsatellite markers for genotyping the A and B genomes of wheat. Theoretical and Applied Genetics.

[B12651853] Evanno G., Regnaut S., Goudet J. (2005). Detecting the number of clusters of individuals using the software structure: a simulation study. Molecular Ecology.

[B12651867] Excoffier Laurent, Laval Guillaume, Schneider Stefan (2005). Arlequin (version 3.0): An integrated software package for population genetics data analysis. Evolutionary Bioinformatics.

[B12651885] Falk Donald A, Holsinger Kent E (2023). Genetics and conservation of rare plants.

[B12651893] Farjon A. , (2010). A Handbook of the world’s conifers.

[B12651901] Finkeldey R, Hattemer H. H, (2007). Tropical forest genetics.

[B12678548] Finn Robert D., Bateman Alex, Clements Jody, Coggill Penelope, Eberhardt Ruth Y., Eddy Sean R., Heger Andreas, Hetherington Kirstie, Holm Liisa, Mistry Jaina, Sonnhammer Erik L. L., Tate John, Punta Marco (2013). Pfam: the protein families database. Nucleic Acids Research.

[B12678767] Geng Qi-Fang, Liu Jun, Sun Lin, Liu Hong, Ou-Yang Yan, Cai Ying, Tang Xin-Sheng, Zhang Hong-Wei, Wang Zhong-Sheng, An Shu-Qing (2015). Development and characterization of polymorphic microsatellite markers (SSRs) for an endemic plant, *Pseudolarixamabilis* (Nelson) Rehd. (Pinaceae). Molecules.

[B12678510] Götz Jeremias, Leinemann Ludger, Gailing Oliver, Hardtke André, Caré Oliver (2024). Development of a highly polymorphic chloroplast SSR set in *Abiesgrandis* with transferability to other conifer species-A promising toolkit for gene flow investigations. Ecology and Evolution.

[B12651940] Grabherr Manfred G, Haas Brian J, Yassour Moran, Levin Joshua Z, Thompson Dawn A, Amit Ido, Adiconis Xian, Fan Lin, Raychowdhury Raktima, Zeng Qiandong, Chen Zehua, Mauceli Evan, Hacohen Nir, Gnirke Andreas, Rhind Nicholas, di Palma Federica, Birren Bruce W, Nusbaum Chad, Lindblad-Toh Kerstin, Friedman Nir, Regev Aviv (2011). Full-length transcriptome assembly from RNA-Seq data without a reference genome. Nature Biotechnology.

[B12653504] Haji Raja Feroz Ahmad, Bhargava Mili, Akhoon Bashir A., Kumar Amandeep, Brindavanam Narshima B., Verma Vijeshwar (2014). Correlation and functional differentiation between different markers to study the genetic diversity analysis in medicinally important plant *Plumbagozeylanica*. Industrial Crops and Products.

[B12651966] Hamrick J. L., Godt Mary Jo W. (1996). Conservation genetics of endemic plant species. Conservation Genetics.

[B12678604] Hedrick Philip W. (2007). A standarized genetic differentiation measure. Evolution.

[B12678929] Hellmann Jessica J., Pineda-Krch Mario (2007). Constraints and reinforcement on adaptation under climate change: Selection of genetically correlated traits. Biological Conservation.

[B12651993] Huang Hua-Hong, Xu Li-Li, Tong Zai-Kang, Lin Er-Pei, Liu Qing-Po, Cheng Long-Jun, Zhu Mu-Yuan (2012). De novo characterization of the Chinese fir (*Cunninghamialanceolata*) transcriptome and analysis of candidate genes involved in cellulose and lignin biosynthesis. BMC Genomics.

[B12678697] Hu Xian-Ge, Liu Hui, Jin YuQing, Sun Yan-Qiang, Li Yue, Zhao Wei, El-Kassaby Yousry A., Wang Xiao-Ru, Mao Jian-Feng (2016). De novo transcriptome assembly and characterization for the widespread and stress-tolerant conifer *Platycladusorientalis*. PLOS One.

[B12652005] Jain M. (2011). Next-generation sequencing technologies for gene expression profiling in plants. Briefings in Functional Genomics.

[B12678632] Jakobsson Mattias, Rosenberg Noah A. (2007). Clumpp: a cluster matching and permutation program for dealing with label switching and multimodality in analysis of population structure. Bioinformatics.

[B12678650] Jombart Thibaut, Devillard Sébastien, Balloux François (2010). Discriminant analysis of principal components: a new method for the analysis of genetically structured populations. BMC Genetics.

[B12678576] Kalinowski S. T., Taper M. L., Marshall T. C. (2007). Revising how the computer program cervus accommodates genotyping error increases success in paternity assignment. Molecular Ecology.

[B12652024] Kanehisa M. (2004). The KEGG resource for deciphering the genome. Nucleic Acids Research.

[B12653562] Kantety Ramesh V, La Rota Mauricio, Matthews David E, Sorrells Mark E (2002). Data mining for simple sequence repeats in expressed sequence tags from barley, maize, rice, sorghum and wheat.. Plant molecular biology.

[B12653614] Kim Kyung Seok, Sappington Thomas W. (2013). Microsatellite data analysis for population genetics. Methods in Molecular Biology.

[B12652044] Koonin Eugene V, Fedorova Natalie D, Jackson John D, Jacobs Aviva R, Krylov Dmitri M, Makarova Kira S, Mazumder Raja, Mekhedov Sergei L, Nikolskaya Anastasia N, Rao B Sridhar, Rogozin Igor B, Smirnov Sergei, Sorokin Alexander V, Sverdlov Alexander V, Vasudevan Sona, Wolf Yuri I, Yin Jodie J, Natale Darren A (2004). A comprehensive evolutionary classification of proteins encoded in complete eukaryotic genomes. Genome Biology.

[B12653515] Kumpatla Siva P, Mukhopadhyay Snehasis (2005). Mining and survey of simple sequence repeats in expressed sequence tags of dicotyledonous species. Genome.

[B12678870] Law Julie A., Jacobsen Steven E. (2010). Establishing, maintaining and modifying DNA methylation patterns in plants and animals. Nature Reviews Genetics.

[B12678613] Legendre P., Fortin M. J. (2010). Comparison of the mantel test and alternative approaches for detecting complex multivariate relationships in the spatial analysis of genetic data. Molecular Ecology Resources.

[B12678389] Liang W. Y., (2010). The cutting propagation technique and afforestation experiment of *Cunninghamiakonishii*. Subtropical Agriculture Research.

[B12652100] Lin Erpei, Zhuang Hebi, Yu Jinjian, Liu Xueyu, Huang Huahong, Zhu Muyuan, Tong Zaikang (2020). Genome survey of Chinese fir (*Cunninghamialanceolata*): Identification of genomic SSRs and demonstration of their utility in genetic diversity analysis. Scientific Reports.

[B12652112] Liu Jie, Gao Lian‐Ming, Li De‐Zhu, Zhang De‐Quan, Möller Michael (2011). Cross‐species amplification and development of new microsatellite loci for *Taxuswallichiana* (Taxaceae). American Journal of Botany.

[B12652087] Li Xin-Yu, Lin Xue-Ying, Ruhsam Markus, Chen Lu, Wu Xing-Tong, Wang Min-qiu, Thomas Philip I., Wen Ya-Feng (2019). Development of microsatellite markers for the critically endangered conifer *Glyptostrobuspensilis* (Cupressaceae) using transcriptome data. Silvae Genetica.

[B12652076] Li Xiang, Liu Xiaoting, Wei Jiatong, Li Yan, Tigabu Mulualem, Zhao Xiyang (2020). Development and transferability of EST-SSR markers for *Pinuskoraiensis* from cold-stressed transcriptome through illumina sequencing. Genes.

[B12653600] Luro François L, Costantino Gilles, Terol Javier, Argout Xavier, Allario Thierry, Wincker Patrick, Talon Manuel, Ollitrault Patrick, Morillon Raphael (2008). Transferability of the EST-SSRs developed on Nules clementine (*Citrusclementina* Hort ex Tan) to other Citrus species and their effectiveness for genetic mapping. BMC Genomics.

[B12652132] Most, Vast (2024). Red data book of Vietnam.

[B12652154] Murawski Darlyne A., Dayanandan Bama, Bawa Kamaljit S. (1994). Outcrossing rates of two endemic *Shorea* species from Sri Lankan tropical rain forests. Biotropica.

[B12652288] Nguyen Tam Minh, Vu Duy Dinh, Nguyen Duc Minh, Dang Hien Phan, Phan Long Ke, Bui Phuong Xuan (2020). Microsatellite analysis reveals genetic diversity of the endangered species *Dipterocarpusdyeri*. Journal of Forest Research.

[B12652299] Peakall Rod, Smouse Peter E. (2012). GenAlEx 6.5: genetic analysis in Excel. Population genetic software for teaching and research-an update. Bioinformatics.

[B12652308] Pertea Geo, Huang Xiaoqiu, Liang Feng, Antonescu Valentin, Sultana Razvan, Karamycheva Svetlana, Lee Yuandan, White Joseph, Cheung Foo, Parvizi Babak, Tsai Jennifer, Quackenbush John (2003). TIGR Gene Indices clustering tools (TGICL): a softwaresystem for fast clustering of large EST datasets. Bioinformatics.

[B12652547] Pham Mai Phuong, Vu Dinh Duy (2022). Genetic diversity and populations structure of the endangered species *Cunninghamiakonishii* Hayata in Vietnam using SSR molecular markers. Research Journal of Biotechnology.

[B12678378] Phan K. L., Pham V. T., Phan K. L., Regalado J., Averyanov L. V., Maslin B. (2017). Native conifers of Vietnam - a review. Pakistan Journal of Botany.

[B12678855] Pinosio S., González‐Martínez S. C., Bagnoli F., Cattonaro F., Grivet D., Marroni F., Lorenzo Z., Pausas J. G., Verdú M., Vendramin G. G. (2014). First insights into the transcriptome and development of new genomic tools of a widespread circum‐Mediterranean tree species, *Pinushalepensis* Mill. Molecular Ecology Resources.

[B12652325] Piry S, Luikart G, Cornuet J-M (1999). Computer note. Botteneck: a computer program for detecting recent reductions in the effective size using allele frequency data. Journal of Heredity.

[B12652334] Postolache Dragos, Leonarduzzi Cristina, Piotti Andrea, Spanu Ilaria, Roig Anne, Fady Bruno, Roschanski Anna, Liepelt Sascha, Vendramin Giovanni Giuseppe (2013). Transcriptome versus genomic microsatellite markers: highly informative multiplexes for genotyping *Abiesalba* Mill. and Congeneric Species. Plant Molecular Biology Reporter.

[B12652348] Pritchard Jonathan K, Stephens Matthew, Donnelly Peter (2000). Inference of population structure using multilocus genotype data. Genetics.

[B12652366] Qin Hantao, Yang Guoqian, Provan Jim, Liu Jie, Gao Lianming (2017). Using Mi ddRAD-seq data to develop polymorphic microsatellite markers for an endangered yew species. Plant Diversity.

[B12678880] Ranade Sonali Sachin, Lin Yao-Cheng, Zuccolo Andrea, Van de Peer Yves, García-Gil María del Rosario (2014). Comparative in silicoanalysis of EST-SSRs in angiosperm and gymnosperm tree genera. BMC Plant Biology.

[B12678641] Rosenberg Noah A. (2003). Distruct: a program for the graphical display of population structure. Molecular Ecology Notes.

[B12652401] Ruan Xiaoxian, Wang Zhen, Wang Ting, Su Yingjuan (2019). Characterization and application of EST-SSR markers developed from the transcriptome of *Amentotaxusargotaenia* (Taxaceae), a relict vulnerable conifer. Frontiers in Genetics.

[B12678743] Rungis Dainis, Bérubé Yanik, Zhang Jun, Ralph Steven, Ritland Carol E., Ellis Brian E., Douglas Carl, Bohlmann J�rg, Ritland Kermit (2004). Robust simple sequence repeat markers for spruce (*Picea* spp.) from expressed sequence tags. Theoretical and Applied Genetics.

[B12652478] Slatkin Montgomery (1987). Gene flow and the geographic structure of natural populations. Science.

[B12653470] Squirrell J., Hollingsworth P. M., Woodhead M., Russell J., Lowe A. J., Gibby M., Powell W. (2003). How much effort is required to isolate nuclear microsatellites from plants?. Molecular Ecology.

[B12652510] Srivastava Surabhi, Avvaru Akshay Kumar, Sowpati Divya Tej, Mishra Rakesh K. (2019). Patterns of microsatellite distribution across eukaryotic genomes. BMC Genomics.

[B12653641] Stàgel Anikò, Portis Ezio, Toppino Laura, Rotino Giuseppe Leonardo, Lanteri Sergio (2008). Gene-based microsatellite development for mapping and phylogeny studies in eggplant. BMC Genomics.

[B13296244] Takezaki N, Nei M, Tamura K (2010). POPTREE2: software for constructing population trees from allele frequency data and computing other population statistics with Windows interface. Mol. Evol..

[B12652611] Thomas P, Yang Y (2013). Cunninghamiakonishii. 2013: e.T31258A2802692.

[B12678445] Tran H. T., Ophélie B., Tran M. H., Do T. M., Phan K. L., Nguyen T. T.N., Félix T., Joseph C., Ange B. (2015). Chemical composition of the essential oil from *Cunninghamiakonishii* Hayata growing wild in Vietnam. American Journal of Essential Oils and Natural Products.

[B12678806] Ueno Saneyoshi, Moriguchi Yoshinari, Uchiyama Kentaro, Ujino-Ihara Tokuko, Futamura Norihiro, Sakurai Tetsuya, Shinohara Kenji, Tsumura Yoshihiko (2012). A second generation framework for the analysis of microsatellites in expressed sequence tags and the development of EST-SSR markers for a conifer, *Cryptomeriajaponica*. BMC Genomics.

[B12678967] Untergasser Andreas, Cutcutache Ioana, Koressaar Triinu, Ye Jian, Faircloth Brant C., Remm Maido, Rozen Steven G. (2012). Primer3-new capabilities and interfaces. Nucleic Acids Research.

[B12678567] Van Oosterhout C, Hutchinson WF, Wills DPM, Shipley P (2004). microchecker: software for identifying and correcting genotyping errors in microsatellite data. Molecular Ecology Notes.

[B12652649] Varshney Rajeev K., Graner Andreas, Sorrells Mark E. (2005). Genic microsatellite markers in plants: features and applications. Trends in Biotechnology.

[B12652717] Vlk David, Řepková Jana (2017). Application of next-generation sequencing in plant breeding. Czech Journal of Genetics and Plant Breeding.

[B12678819] Wegrzyn Jill L, Liechty John D, Stevens Kristian A, Wu Le-Shin, Loopstra Carol A, Vasquez-Gross Hans A, Dougherty William M, Lin Brian Y, Zieve Jacob J, Martínez-García Pedro J, Holt Carson, Yandell Mark, Zimin Aleksey V, Yorke James A, Crepeau Marc W, Puiu Daniela, Salzberg Steven L, de Jong Pieter J, Mockaitis Keithanne, Main Doreen, Langley Charles H, Neale David B (2014). Unique features of the loblolly pine (*Pinustaeda* L.) megagenome revealed through sequence annotation. Genetics.

[B12678594] Weir B. S., Cockerham C. Clark (1984). Estimating F-Statistics for the analysis of population structure. Evolution.

[B12652852] Wen Y., Ueno S., Han W. (2017). Development and characterization of 28 polymorphic EST-SSR markers for *Cunninghamialanceolata* (Taxodiaceae) based on transcriptome sequences. Silvae Genetica.

[B12678948] White T. L.,, Adams W. T.,, Neale D. B., (2007). Forest genetics.

[B12652917] Wu X., Wen Y., Ueno S., Tsumura Y. (2017). Development and characterization of EST-SSR markers for *Taxusmairei* (Taxaceae) and their transferability across species. Silvae Genetica.

[B12678757] Xiang Xiaoyan, Zhang Zhongxin, Wang Zhigao, Zhang Xiaoping, Wu Ganlin (2015). Transcriptome sequencing and development of EST-SSR markers in *Pinusdabeshanensis*, an endangered conifer endemic to China. Molecular Breeding.

[B12653571] Yu Hong, Li Qi (2008). Exploiting EST databases for the development and characterization of EST-SSRs in the pacific oyster (*Crassostreagigas*). Journal of Heredity.

[B12678728] Zalapa Juan E, Cuevas Hugo, Zhu Huayu, Steffan Shawn, Senalik Douglas, Zeldin Eric, McCown Brent, Harbut Rebecca, Simon Philipp (2011). Using next-generation sequencing approaches to isolate simple sequence repeat (SSR) loci in the plant sciences.. American journal of botany.

[B12653461] Zane L, Bargelloni L, Patarnello T (2002). Strategies for microsatellite isolation: a review. Molecular Ecology.

[B12652953] Zeng Jun, Chen Jie, Kou Yixuan, Wang Yujin (2018). Application of EST-SSR markers developed from the transcriptome of *Torreyagrandis* (Taxaceae), a threatened nut-yielding conifer tree. PeerJ.

[B12652988] Zhang Cuiping, Wu Zhonglan, Jiang Xinqiang, Li Wei, Lu Yizeng, Wang Kuiling (2021). De novo transcriptomic analysis and identification of EST-SSR markers in *Stephanandraincisa*. Scientific Reports.

[B12653037] Zhu Lin, Lou Anru (2012). Development and characterization of nine highly polymorphic microsatellite primers for *Platycladusorientalis* (Cupressaceae). American Journal of Botany.

