# Supplementary figures and images for: Transcriptome characterisation and population genetics of Cunninghamiakonishii Hayata – An endangered gymnosperm and implication for its conservation in Vietnam
Source: Biodivers Data J. 2025 Jul 18;13:e153663. doi: 10.3897/BDJ.13.e153663 (PMC12296577; doi:10.3897/BDJ.13.e153663)

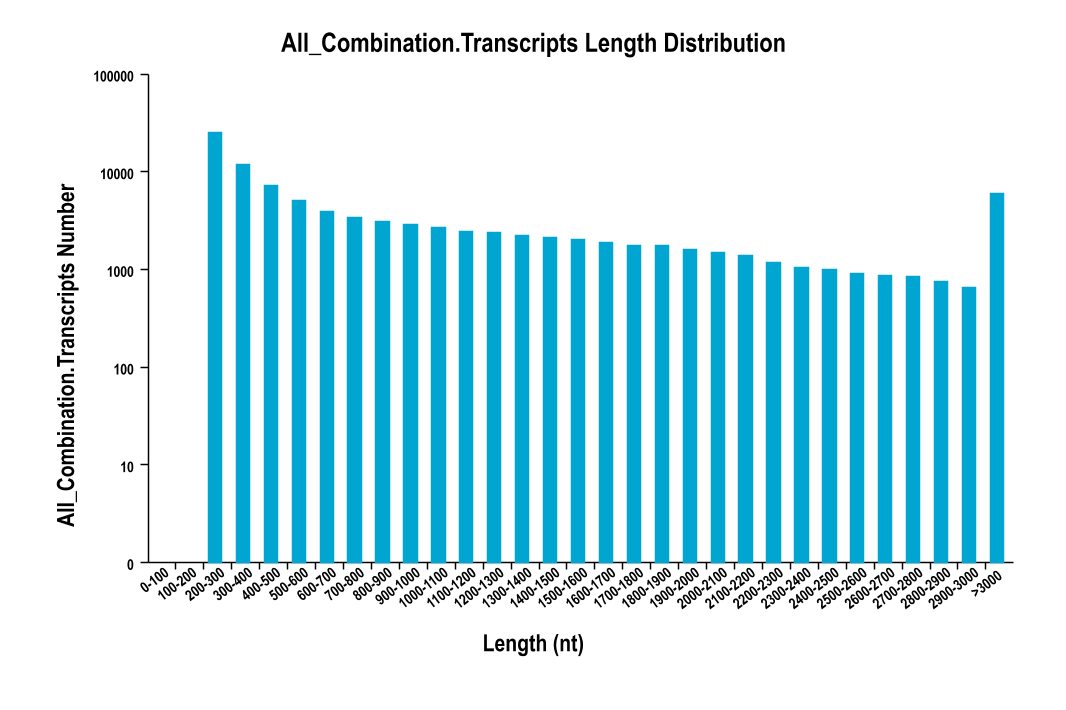

Supplement: Supplementary material 1 — Length distribution of assembly transcript [file bdj-13-e153663-s001.png]

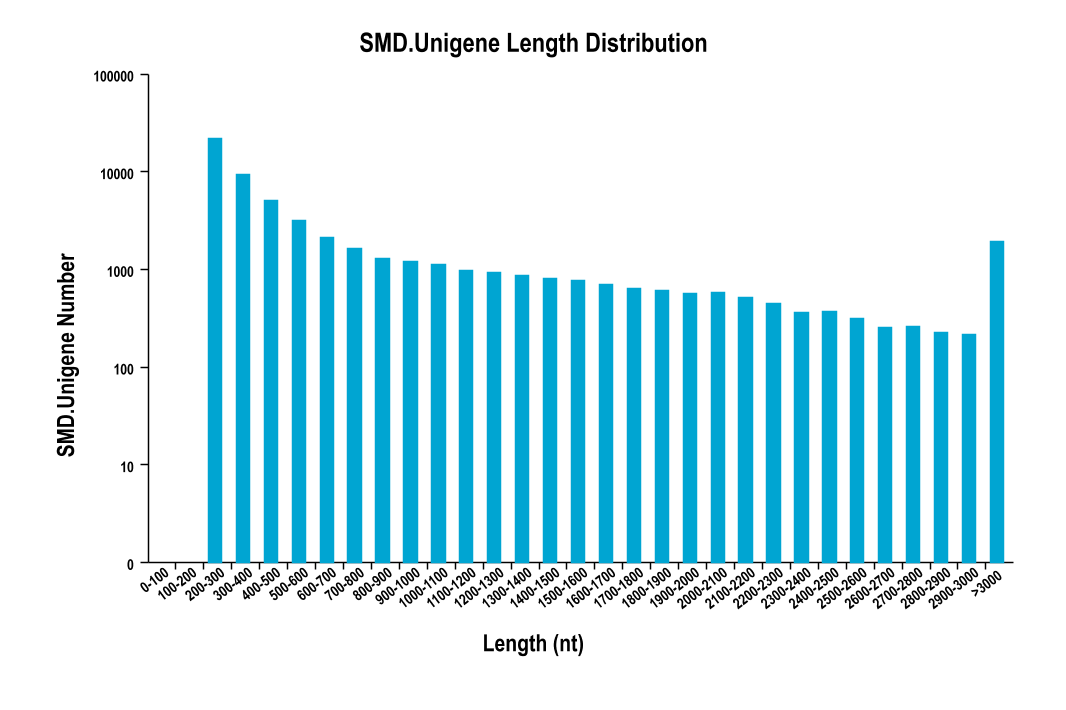

Supplement: Supplementary material 2 — Length distribution of assembly unigenes [file bdj-13-e153663-s002.png]

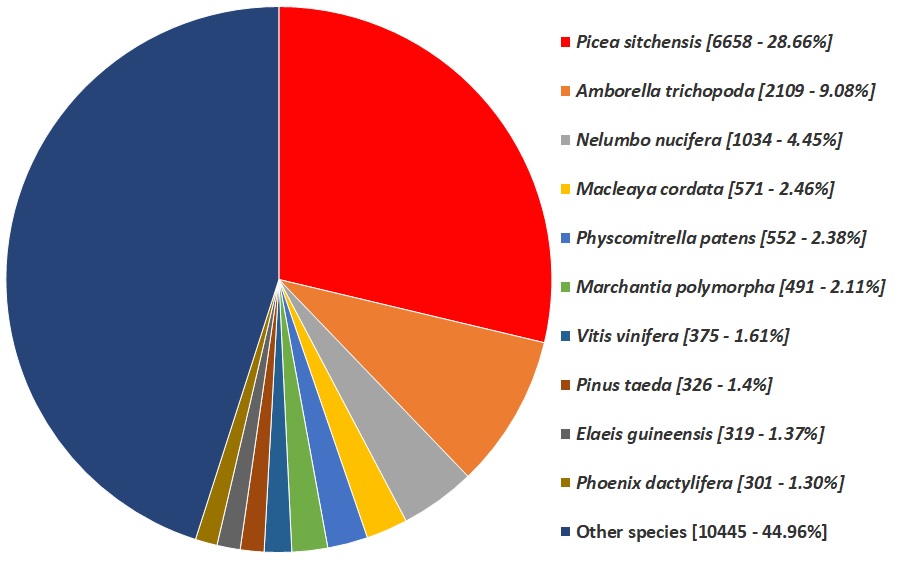

Supplement: Supplementary material 3 — Distribution of species search of unigenes [file bdj-13-e153663-s003.jpg]

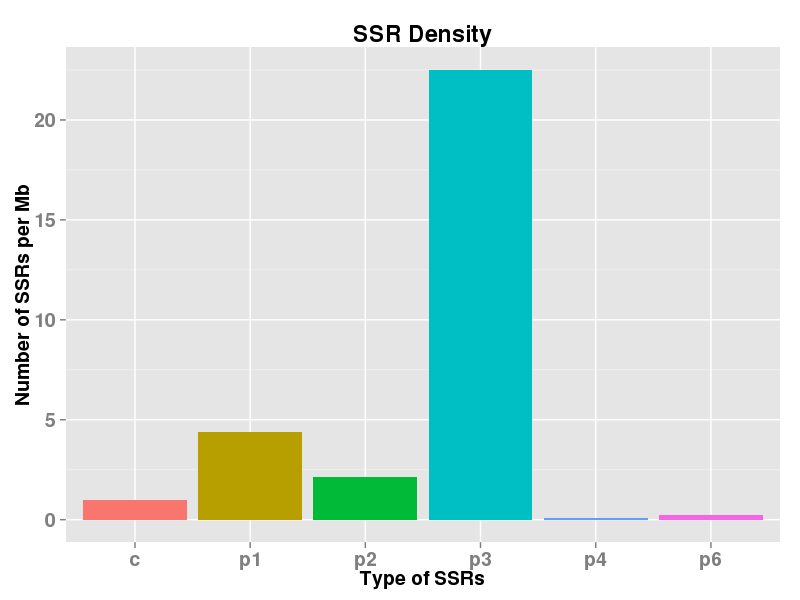

Supplement: Supplementary material 4 — Distribution of different repeat type classes [file bdj-13-e153663-s004.png]

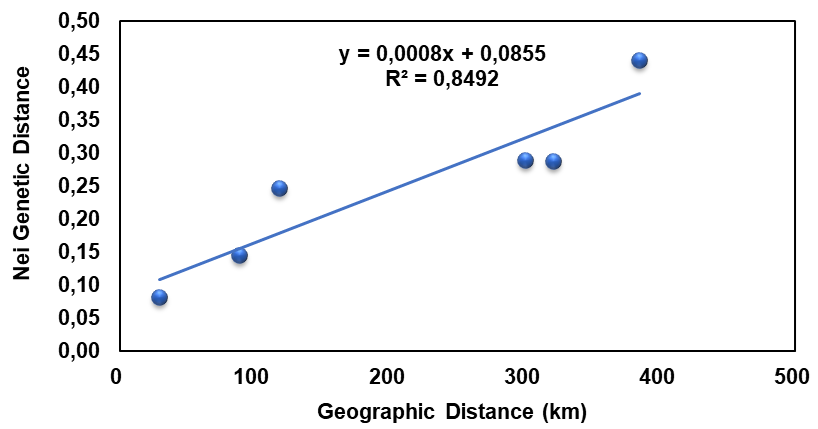

Supplement: Supplementary material 5 — Mantel test of genetic distance and geographical distance [file bdj-13-e153663-s005.png]
