# Supplementary material for: Transcriptome characterisation and population genetics of Cunninghamiakonishii Hayata – An endangered gymnosperm and implication for its conservation in Vietnam
Source: Biodivers Data J. 2025 Jul 18;13:e153663. doi: 10.3897/BDJ.13.e153663 (PMC12296577; doi:10.3897/BDJ.13.e153663)
Supplement: Supplementary material 6 — Table S1. Sampling location [file bdj-13-e153663-s006.docx]

| **Table S1.** Sampling location *C. konishii* from Vietnam in the present study | | | | | |
| --- | --- | --- | --- | --- | --- |
| **Population code** | **Location** | **Altitude (meters)** | **Latitude (N)** | **Longitude (E)** | **Sample size** |
| **HSP** | Hoang Su Phi Nature Reserve, Hoang Su Phi District, Ha Giang province | 1337 | 22^o^39’49.6” | 104^o^45’35.1” | 27 |
| **XL** | Xuan Lien Nature Reserve, Thanh Hoa province | 1255 | 19^o^58’31.7” | 104^o^59’43.0” | 34 |
| **PH** | Pu Hoat Nature Reserve, Que Phong District, Nghe An province | 1366 | 19^o^46’55.5” | 104^o^48’22.4” | 35 |
