# Supplementary material for: Transcriptome characterisation and population genetics of Cunninghamiakonishii Hayata – An endangered gymnosperm and implication for its conservation in Vietnam
Source: Biodivers Data J. 2025 Jul 18;13:e153663. doi: 10.3897/BDJ.13.e153663 (PMC12296577; doi:10.3897/BDJ.13.e153663)
Supplement: Supplementary material 7 — Table S2. The main identified essential oil biosynthetic genes [file bdj-13-e153663-s007.docx]

| **Table S2.** The main identified essential oil biosynthetic genes from *C. konishii* unigenes | | | |
| --- | --- | --- | --- |
| **No** | **Gene ID** | **Candidate genes** | **Number unigenes** |
| 1 | ACC | K11262 acetyl-CoA carboxylase / biotin carboxylase 1 [EC:6.4.1.2 6.3.4.14] | 7 |
| 2 | EAR | K00208 enoyl-[acyl-carrier protein] reductase I [EC:1.3.1.9 1.3.1.10] | 66 |
| 3 | FatA | K10782 fatty acyl-ACP thioesterase A [EC:3.1.2.14] | 1 |
| 4 | FatB | K10781 fatty acyl-ACP thioesterase B [EC:3.1.2.14 3.1.2.21] | 3 |
| 5 | HAD | K10527 enoyl-CoA hydratase/3-hydroxyacyl-CoA dehydrogenase [EC:4.2.1.17 1.1.1.35 1.1.1.211] | 6 |
| 6 | KAR | K00059 3-oxoacyl-[acyl-carrier protein] reductase [EC:1.1.1.100] | 6 |
| 7 | KCS | K15397 3-ketoacyl-CoA synthase [EC:2.3.1.199] | 30 |
| 8 | MCMT | K00645 Malonyl-CoA- malonyltransferase [EC:2.3.1.39] | 1 |
| 9 | SAD | K00507 stearoyl-CoA desaturase (Delta-9 desaturase) [EC:1.14.19.1] | 3 |
