# Supplementary material for: Transcriptome characterisation and population genetics of Cunninghamiakonishii Hayata – An endangered gymnosperm and implication for its conservation in Vietnam
Source: Biodivers Data J. 2025 Jul 18;13:e153663. doi: 10.3897/BDJ.13.e153663 (PMC12296577; doi:10.3897/BDJ.13.e153663)
Supplement: Supplementary material 8 — Table S3. Summary of analyses of expressed sequence [file bdj-13-e153663-s008.docx]

| **Table S3.** Summary of analyses of expressed sequence Tag–Simple Sequence repeat (EST-SSRs) in *C. konishii* | | |
| --- | --- | --- |
| **Item** | **Parameters** | **Number** |
| EST-SSR | Total number of sequences examined | 13437 |
|  | Total size of examined sequences (bp) | 27649667 |
|  | Total number of identified SSRs | 2854 |
|  | Number of SSR containing sequences | 2413 |
|  | Number of sequences containing more than 1 SSR | 372 |
|  | Number of SSRs present in compound formation | 98 |
