# Supplementary material for: Transcriptome characterisation and population genetics of Cunninghamiakonishii Hayata – An endangered gymnosperm and implication for its conservation in Vietnam
Source: Biodivers Data J. 2025 Jul 18;13:e153663. doi: 10.3897/BDJ.13.e153663 (PMC12296577; doi:10.3897/BDJ.13.e153663)
Supplement: Supplementary material 9 — Table S4. Frequency distribution of SSRs [file bdj-13-e153663-s009.docx]

| **Table S4.** Frequency distribution of SSRs based on motif types in *C. konishii* transcriptome | | | | | | | | | |
| --- | --- | --- | --- | --- | --- | --- | --- | --- | --- |
| **Microsatellite motif** | **Number of repeats** | | | | | | | **Total** | **Percentage (%)** |
|  | **5** | **6** | **7** | **8** | **9** | **10** | **>10** |  |  |
| A/T | - | - | - | - | - | 724 | 1150 | 1874 | 65.66 |
| C/G | - | - | - | - | - | 4 | 5 | 9 | 0.32 |
| AC/GT | - | 18 | 6 | 3 | 3 | 1 | 5 | 36 | 1.26 |
| AG/CT | - | 61 | 23 | 12 | 4 | 5 | 12 | 117 | 4.10 |
| AT/AT | - | 68 | 21 | 13 | 12 | 9 | 11 | 134 | 4.70 |
| AAC/GTT | 24 | 7 | 7 | 1 |  |  |  | 39 | 1.37 |
| AAG/CTT | 110 | 38 | 18 | 3 |  |  |  | 169 | 5.92 |
| AAT/ATT | 34 | 12 | 6 | 1 |  | 1 |  | 54 | 1.89 |
| ACC/GGT | 21 | 5 | 3 | 1 |  |  |  | 30 | 1.05 |
| ACG/CGT | 7 | 2 |  |  |  |  |  | 9 | 0.32 |
| ACT/AGT | 3 | 1 |  |  |  |  |  | 4 | 0.14 |
| AGC/CTG | 84 | 23 | 14 | 1 |  |  |  | 122 | 4.27 |
| AGG/CCT | 64 | 32 | 16 |  |  |  |  | 112 | 3.92 |
| ATC/ATG | 46 | 24 | 6 | 1 |  |  |  | 77 | 2.70 |
| CCG/CGG | 23 | 11 | 1 | 1 |  |  |  | 36 | 1.26 |
| AAAC/GTTT | 1 |  |  |  |  |  |  | 1 | 0.04 |
| AAAG/CTTT |  | 1 |  |  |  |  |  | 1 | 0.04 |
| AAAT/ATTT | 2 |  |  |  |  |  |  | 2 | 0.07 |
| AAGG/CCTT |  | 3 |  |  |  |  |  | 3 | 0.11 |
| AAGT/ACTT | 1 |  |  |  |  |  |  | 1 | 0.04 |
| AATC/ATTG | 2 |  |  |  |  |  |  | 2 | 0.07 |
| AATG/ATTC | 4 |  |  |  |  |  |  | 4 | 0.14 |
| ACAT/ATGT | 2 |  |  |  |  |  |  | 2 | 0.07 |
| ACCT/AGGT |  | 1 |  |  |  |  |  | 1 | 0.04 |
| AGAT/ATCT | 1 |  |  |  |  |  |  | 1 | 0.04 |
| AGGC/CCTG |  | 1 |  |  |  |  |  | 1 | 0.04 |
| AAATG/ATTTC | 1 |  |  |  |  |  |  | 1 | 0.04 |
| AAGAG/CTCTT | 1 |  |  |  |  |  |  | 1 | 0.04 |
| AATAT/ATATT | 1 |  |  |  |  |  |  | 1 | 0.04 |
| AAAGAC/CTTTGT |  | 1 |  |  |  |  |  | 1 | 0.04 |
| AAAGGC/CCTTTG | 1 |  |  |  |  |  |  | 1 | 0.04 |
| AACACC/GGTGTT |  | 1 |  |  |  |  |  | 1 | 0.04 |
| AACGGG/CCCGTT | 1 |  |  |  |  |  |  | 1 | 0.04 |
| AAGATG/ATCTTC | 1 |  |  |  |  |  |  | 1 | 0.04 |
| AAGGCG/CCTTCG | 1 |  |  |  |  |  |  | 1 | 0.04 |
| AAGGTG/ACCTTC |  | 1 |  |  |  |  |  | 1 | 0.04 |
| ACTGAG/AGTCTC | 1 |  |  |  |  |  |  | 1 | 0.04 |
| ACTGCC/AGTGGC | 1 |  |  |  |  |  |  | 1 | 0.04 |
| AGCCTC/AGGCTG | 1 |  |  |  |  |  |  | 1 | 0.04 |
