# Supplementary material for: Transcriptome characterisation and population genetics of Cunninghamiakonishii Hayata – An endangered gymnosperm and implication for its conservation in Vietnam
Source: Biodivers Data J. 2025 Jul 18;13:e153663. doi: 10.3897/BDJ.13.e153663 (PMC12296577; doi:10.3897/BDJ.13.e153663)
Supplement: Supplementary material 10 — Table S5. Primer sequences, repeat motif, size range of alleles and annealing temperature [file bdj-13-e153663-s010.docx]

| **Table S5.** Primer sequences, repeat motif, size range of alleles, and annealing temperature (Tm) of 10 polymorphic EST-SSR markers developed for *C. konishii* | | | | | |
| --- | --- | --- | --- | --- | --- |
| **Locus** | **Sequence of primer (5'–3' )** | **Motif type** | **Size (bp)** | **Ta (°C)** | **Genbank** |
| MP01 | F:AGACGCCTTTTCTCCTGCTC  R:CACCTCCTTCGCAATCATCT | (CCGTTC)5 | 274 | 55 | MW366236 |
| **MP11** | F:ATAGGGGTTCTTGGGCAGAG  R:GGTCACGCATGTTAAGGGAT | (GGAA)6 | 202 | 55 | MW366246 |
| **MP13** | F:AAATTACCGCTGCACGAAAC  R:AGCTTACGACCTCCATCCAG | (TTCA)5 | 176 | 54 | MW366248 |
| **MP15** | F:AGGAGCCACAAGAGAGGAGATT  R:CAGAAGGTCCAAGAACCAAGAG | (TTTA)5 | 277 | 55 | MW366250 |
| **MP16** | F:ACAGCCTTTTGATGAGCTATGTC  R:CCTTGTTGCTCTTTCTTTCTGC | (GAAG)6 | 154 | 55 | MW366251 |
| **MP17** | F:AAGTTGTGGTGCTCGCTCA  R:CCGTGTGCGTGTGTAACTATCT | (TTCT)6 | 231 | 55 | MW366253 |
| **MP18** | F:AAGTGTCTTAGGGCTGAGGCTAT  R:GGGAGCATTTGTAGATGGAACT | (TTCA)5 | 271 | 53 | MW366254 |
| **MP21** | F:GCTACTGGTGCTGCTGATGA  R:GAAGCCAAGTTCTCCACGAA | (ATA)7 | 180 | 55 | MW366257 |
| **MP28** | F:GAAGAGATGCAGAGGATGCC  R:AGCACTTTAGATCGCCTCCA | (GTG)7 | 150 | 53 | MW366264 |
| **MP30** | F:TGTGAGTCGCCTAAACCACA  R:GCACTTTCAGCAGCCTCTCT | (ATC)7 | 239 | 55 | MW366266 |
