# Supplementary material for: Transcriptome characterisation and population genetics of Cunninghamiakonishii Hayata – An endangered gymnosperm and implication for its conservation in Vietnam
Source: Biodivers Data J. 2025 Jul 18;13:e153663. doi: 10.3897/BDJ.13.e153663 (PMC12296577; doi:10.3897/BDJ.13.e153663)
Supplement: Supplementary material 11 — Table S6. Pairwise genetic differentiation (FST) between populations [file bdj-13-e153663-s011.docx]

| **Table S6.** Pairwise genetic differentiation (F_ST_) between populations for *C. konishii* species. | | | | |
| --- | --- | --- | --- | --- |
|  | **XL** | **HSP** | **PH** |  |
| **XL** | 0.000 |  |  | **XL** |
| **HSP** | 0.020^*^ | 0.000 |  | **HSP** |
| **PH** | 0.034^**^ | 0.014 | 0.000 | **PH** |
|  | **XL** | **HSP** | **PH** |  |
