# Supplementary material for: Transcriptome characterisation and population genetics of Cunninghamiakonishii Hayata – An endangered gymnosperm and implication for its conservation in Vietnam
Source: Biodivers Data J. 2025 Jul 18;13:e153663. doi: 10.3897/BDJ.13.e153663 (PMC12296577; doi:10.3897/BDJ.13.e153663)
Supplement: Supplementary material 12 — Table S7. Number of individuals for each population assigned [file bdj-13-e153663-s012.docx]

| **Table S7**. Number of individuals for each population assigned. Each cluster was obtained from DAPC without prior information. | | | |
| --- | --- | --- | --- |
| **Population** | **Cluster 1** | **Cluster 2** | **Cluster 3** |
| **XL** | 12 | 7 | 15 |
| **HSP** | 7 | 11 | 9 |
| **PH** | 10 | 16 | 9 |
