# Supplementary material for: Transcriptome characterisation and population genetics of Cunninghamiakonishii Hayata – An endangered gymnosperm and implication for its conservation in Vietnam
Source: Biodivers Data J. 2025 Jul 18;13:e153663. doi: 10.3897/BDJ.13.e153663 (PMC12296577; doi:10.3897/BDJ.13.e153663)
Supplement: Supplementary material 13 — Table S8. Percentage of ancestry [file bdj-13-e153663-s013.docx]

| **Table S8.** Percentage of ancestry for three *C. konishii* populations | | | |
| --- | --- | --- | --- |
| **Populations** | **Genetic group** | | |
|  | **I (orange)** | **II (blue)** | **III (purple)** |
| **XL** | 0.473 | 0.160 | 0.368 |
| **HSP** | 0.294 | 0.379 | 0.327 |
| **PH** | 0.160 | 0.477 | 0.363 |
